# Identifying Key Stressors Driving Biological Impairment in Freshwater Streams in the Chesapeake Bay Watershed, USA

**DOI:** 10.1007/s00267-022-01723-7

**Published:** 2022-10-07

**Authors:** Rosemary M. Fanelli, Matthew J. Cashman, Aaron J. Porter

**Affiliations:** 1 U.S. Geological Survey, South Atlantic Water Science Center, Raleigh, NC USA; 2 U.S. Geological Survey, Maryland-D.C.-Delaware Water Science Center, Baltimore, MD USA; 3 U.S. Geological Survey, Virginia-West Virginia Water Science Center, Richmond, VA USA

**Keywords:** Biological assessment, Stressor, Macroinvertebrate, Water quality, Geomorphology, Toxic contaminants

## Abstract

Biological communities in freshwater streams are often impaired by multiple stressors (e.g., flow or water quality) originating from anthropogenic activities such as urbanization, agriculture, or energy extraction. Restoration efforts in the Chesapeake Bay watershed, USA seek to improve biological conditions in 10% of freshwater tributaries and to protect the biological integrity of existing healthy watersheds. To achieve these goals, resource managers need to better understand which stressors are most likely driving biological impairment. Our study addressed this knowledge gap through two approaches: 1) reviewing and synthesizing published multi-stressor studies, and 2) examining 303(d) listed impairments linked to biological impairment as identified by jurisdiction regulatory agencies (the states within the watershed and the District of Columbia). Results identified geomorphology (i.e., physical habitat), salinity, and toxic contaminants as important for explaining variability in benthic community metrics in the literature review. Geomorphology (i.e., physical habitat and sediment), salinity, and nutrients were the most reported stressors in the jurisdictional impairment analysis. Salinity is likely a major stressor in urban and mining settings, whereas geomorphology was commonly reported in agricultural settings. Toxic contaminants, such as pesticides, were rarely measured; more research is needed to quantify the extent of their effects in the region. Flow alteration was also highlighted as an important urban stressor in the literature review but was rarely measured in the literature or reported by jurisdictions as a cause of impairment. These results can be used to prioritize stressor monitoring by managers, and to improve stressor identification methods for identifying causes of biological impairment.

## Introduction

**Fig. 1:**
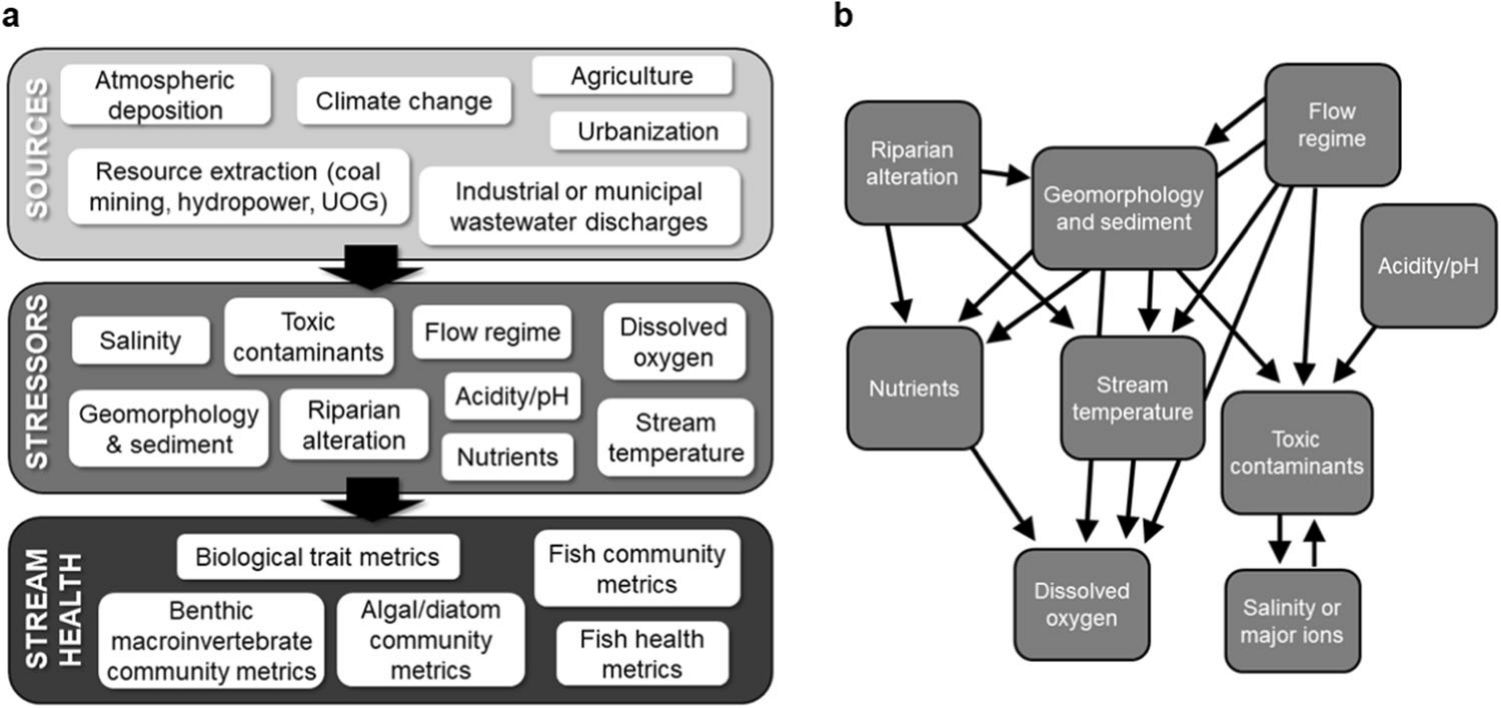
Conceptual diagrams of (**a**) relations between sources (i.e., human activities), in-stream stressors generated from those sources, and measures of stream health; and (**b**) interactions between stressor groups that are often listed as causes of biological impairment. Arrows denote when one stressor type can affect, or influence, another stressor’s condition. Note that riparian alteration is considered a near-stream stressor in this study. UOG Unconventional oil and gas extraction. Presentation of sources, stressors, and stream health relations are based on information provided in US EPA 2017

Freshwater stream ecosystems are subjected to the effects of myriad anthropogenic activities, such as urbanization, climate change, energy extraction, or point-source pollution (Orr et al. 2020; Birk et al. 2020; Waite et al. 2021). These activities (herein referred to as sources) often change conditions in streams and rivers, including: flow or thermal regimes; increased nutrient, sediment, or contaminant loads; or modified in-stream habitat (herein referred to collectively as stressors). These stressors may lead to shifts in the structure and function of one or more biological communities or other measures of stream biological health (Ormerod et al. 2010; Strayer and Dudgeon 2010; Fig. 1a). Multiple stressors may originate from a source, as is the case with the urban stream syndrome (Walsh et al. 2005), making it difficult to identify the primary stressor or stressors causing biological impairment. Often, multiple sources may exist in the same area as well (for example, both point source and non-point sources of water quality pollution in urban settings), further complicating accurate causal attribution to biological impairment. Widespread biological impairment has been documented nationally from these anthropogenic sources and their associated stressors. For example, 44% and 37% of assessed stream reaches had poor benthic or fish community metrics, respectively, in the latest National Streams and Rivers Assessment (U.S. EPA 2020). Natural resource managers need more information on stressors driving biological impairment to prioritize management, restoration, and conservation efforts.

State agencies in the United States conduct regulatory ecological assessments to identify and report causes of biological impairment on their jurisdictional 303(d) impaired waters lists, as required for compliance with the Clean Water Act (1972). These regulatory assessments cover many or most jurisdictional waters, which is helpful for understanding the prevalence of stressors across a region. For example, sediment was identified as the top stressor in a recent U.S. national-scale synthesis of 303(d) impaired waters lists for streams whose benthic macroinvertebrate communities were impaired (Governor et al. 2017). The large spatial footprint of these regulatory assessment programs, however, may limit the depth and robustness of monitoring at individual sites to characterize and identify key stressors (for example, the number of samples taken to define stressor conditions). By contrast, research-focused ecological assessments may have the financial resources and/or smaller spatial scale to apply novel techniques for stressor identification or to quantify stressor conditions more robustly (Suter and Cormier 2016). Stressor groups are often tightly coupled (Fig. 1b), so there is a risk for attributing biological impairment to a proxy stressor, which is a stressor related to the actual primary stressor (e.g., nutrients vs. dissolved oxygen). Moreover, the incomplete consideration of all potential stressors could risk attributing biological impairment to a co-occurring stressor (nutrients vs. pesticides in agricultural areas). To develop a complete understanding of key stressors affecting biological impairment in a region, both regulatory assessments and research-focused assessments must be considered.

The Chesapeake Bay (CB) watershed, located in the mid-Atlantic region, USA, has one of the most intensive ecosystem restoration programs in the United States (US EPA 2010). The health and biological integrity of freshwater tributaries within the watershed is an important focus of this restoration effort (Chesapeake Bay Program 2020). The restoration program includes a goal to improve the health and function of 10% of freshwater tributaries in the 64,020 mi^2^ watershed (Chesapeake Bay Program 2020), and watershed and stream restoration is anticipated to be a significant contributor to achieving that goal. However, research on such practices has not documented expected improvements in biological conditions (Fanelli et al. 2019, Violin et al. 2011). One potential explanation is that restoration and management efforts may not be targeting the primary stressors that are affecting biological communities (Palmer et al. 2010). The lack of observed improvements may highlight a key knowledge gap regarding the stressors primarily responsible for biological impairment in freshwater streams.

To support the needs of regional stakeholders, this synthesis sought to identify the stressors most reported to be causing biological impairment in freshwater stream ecosystems in the CB watershed. We leveraged two sources of information to support this synthesis: 1) published studies examining the effects of multi-stressor conditions (herein referred to as the literature review); and 2) regulatory-listed impairment information for the seven jurisdictions (Delaware [DE], District of Columbia [DC], Maryland [MD], Pennsylvania [PA], New York [NY], Virginia [VA], and West Virginia [WV]) in the CB watershed (herein referred to as the jurisdictional impairment analysis). This study uses benthic macroinvertebrate community metrics to define stream health, since benthic macroinvertebrate communities have long been used as an indicator of overall biological integrity (Karr 1991; Suter and Cormier 2015). However, we also reflect on the literature that reported other ecological endpoints, such as fish or algal communities, since other endpoints may respond differently to the same suite of stressors in a multi-stressor environment (Waite et al. 2021). In this synthesis, we summarize the results from each resource (jurisdictional vs. literature) and discuss the similarities and differences in those findings. We also identify gaps in the scientific literature for which more work is needed to understand certain stressors or drivers and discuss needs to better understand regional patterns in stressors driving in-stream biological impairment. Finally, we discuss the limitations of our analysis and critical next steps to improve our understanding of stressors and their relations to stream health.

## Methods

This review and synthesis focused on nine major (e.g., coarse) stressor groups (Fig. 1b). The U.S. EPA’s general organizational structure for defining stressor categories was used for this study to harmonize results across the literature review and jurisdictional impairment analysis (Cormier et al. 2003). In addition, two stressor groups (geomorphology and toxic contaminants) are composed of finer-scale categories, which are discussed in the results as well. Physical habitat characteristics and suspended sediment dynamics, grouped together under the stressor group “geomorphology,” are the result of local-scale interactions among flow, sediment, channel morphology, and vegetation and are sometimes collectively referred to as hydromorphology (Orr et al. 2008). Given this interaction, we report them as a single stressor group in most of the results. Physical habitat includes metrics describing small-scale bed habitat condition (e.g., embeddedness), sediment/hydraulic combinations (e.g., riffle quality), or local-scale hydraulics and flow (e.g., water depth; Table 1). The “flow” stressor category differs from the geomorphology category in that it describes the stream flow regime, which is shaped by factors acting at larger spatial scales. The toxic contaminants stressor category was also further defined into finer categories, including mercury, metals, pesticides, and other organic contaminants (Table 1). We also report riparian conditions as an additional stressor category since it is often included as a stressor in multi-stressor studies and is considered as a possible stressor in regulatory assessments.

**Table 1:** Major (i.e., coarse) stressor categories and reported stressor measurements as defined in the 33 studies used in the literature review meta-analysis

Stressor category		How stressor was reported in the literature
Coarse	Fine	
Acidity and pH		Discrete measures of pH; acid neutralizing capacity (ANC)
Dissolved oxygen		Single measures of dissolved oxygen; mean or minimum concentrations based on multiple measurements
Flow		Duration or frequency of high flows based on continuous stage or discharge monitoring; continuous monitoring of flow or velocity during flume experiments; a single measure of discharge at a site; estimated bankfull flow; estimated discharge using Area Ratio method; qualitative score from habitat assessment describing flow conditions
Nutrients		Single discrete measures, or median or mean of multiple discrete measures of total nitrogen, ammonium, nitrate, total phosphorus, or soluble reactive phosphorus
Riparian conditions		Estimates of shade or light in channel; percent of riparian zone as forest, agricultural or row crop land use; width of riparian vegetated zone
Salinity or major ions		Single measures or mean, median, or maximum of multiple measures of conductivity/specific conductance; single or multiple measures of chloride, sulfate, calcium, magnesium, or potassium concentrations; discrete measures of water hardness
Stream temperature		Maximum daily temperatures from high-frequency monitoring; single discrete measures, or mean of multiple discrete measures of stream temperature; annual degree days of water temperature based on high-frequency monitoring
Geomorphology	Physical habitat	The addition of fine sediment during flume experiments; measures of percent fines; particle size distribution or particle size variability from cross-sectional surveys; measures of depth or velocity in cross-sectional transects; qualitative scores reflecting overall habitat quality, riffle quality, channel erosion, average amounts of fine sediment, sedimentation, channel alteration or in-stream habitat cover
	Sediment	Single measures or mean of multiple discrete measures of suspended sediment concentrations or total suspended sediment
Toxic contaminants	Mercury	Discrete measures of mercury in streambed material
	Metals	Discrete measures of dissolved iron, selenium, or manganese; probable effect concentration (PEC) quotient for trace elements
	Pesticides	Concentrations of pesticides (insecticides, herbicides or fungicides) from POCIS samplers; median concentrations from multiple discrete samples of pesticides; likely effects benchmark (LEB) quotient for current-use pesticides; probable effect concentration (PEC) quotient for legacy organochlorine pesticides; number of pesticides detected through sampling
	Other	Probable effect concentration (PEC) quotient for polycyclic aromatic hydrocarbons (PAHs); toxic units (TUs) for surfactants generated for community-level responses using single species acute and/or chronic toxicity values

Categories follow EPA organizational structure. Geomorphology and toxic contaminants are further broken into finer categories for clarity

### Methods for the Literature Review and Meta-analysis

We focused our literature review on studies examining the effects of multiple stressors on freshwater benthic macroinvertebrate community metrics (herein referred to as biological response metrics), though we also briefly discuss other measures of stream health in the discussion. Studies conducted in wadable (1st–4th order) freshwater streams in the U.S. Mid-Atlantic region (DE, DC, MD, NY, PA, VA, WV), or in neighboring states were prioritized. Exceptions were made for non-field studies (flume, laboratory) and for stressors or sources not commonly studied in the region. Stressors were defined as measures of in-stream or near-stream conditions (for example, nutrient concentrations or measures of riparian alteration), whereas sources were defined as the general anthropogenic activity from which the stressors originated (e.g., agricultural land use, point source discharges, or climate change; Fig. 1a). Relevant studies were identified through predetermined search queries in Elsevier’s Scopus, Web of Science, and Google Scholar. Search queries included the topic of interest (“multi-stressor” or multiple stressor”), the biological response metric (“benthic macroinvertebrates” or some variation), one or more stressors (e.g., “flow,” “nutrients,” “water temperature”), a source (e.g., “urban,” “agriculture,” “mining,” “point sources”), and geographic extent using the state abbreviations listed above. Once the multi-stressor studies were compiled, key information was extracted from each study, including: the study design (e.g., lab, field-based), which stressors were measured and how they were quantified, and how the biological responses were quantified. Study designs were categorized as follows: large spatial gradient studies (i.e., 15+ observational units or OUs), small spatial gradient studies (i.e., 14 or fewer OUs), longitudinal studies with multiple OUs along a single stream or river, and lab-based studies that used flumes or mesocosms.

A subset of the compiled multi-stressor studies was selected for a meta-analysis (see Supplementary Information for details on eligibility criteria). This analysis quantified the frequency at which stressors were measured and reported in the literature as important for explaining patterns in biological response metrics. The meta-analysis was limited to studies for which the effects of multiple stressors on a biological response were considered simultaneously through statistical analyses (e.g., multiple linear regression, random forests, structured equation modeling). Studies must also have reported the relative importance of a stressor explicitly in the results (e.g., variable importance, coefficients or effect size, or a correlation matrix with associated *p*-values). From each study, we compiled which stressors were identified as important for explaining pattens in a biological response variable as determined by each study’s own statistical analysis and expert judgments by the authors. Stressor importance was noted in our database as a binary variable (important or not important), and the number of studies reporting a stressor as important was compared to the number of studies that measured the stressor and expressed as percentages. For studies that reported multiple response variables (for example, both taxa richness and abundance), the results for only one response metric were selected to avoid double-counting studies. We prioritized biological response metrics that represented the most reported metrics in the literature. We filtered studies to present results for the two major land use settings in the CB watershed (agriculture and urban) and for all eligible studies. All other studies in the database that did not fit the criteria for the meta-analysis were retained for inclusion in the discussion.

### Methods for the Jurisdictional Impairment Analysis

For the jurisdictional impairment analysis, we leveraged regulatory information on impaired streams in the region and their associated stressors by accessing U.S. EPA’s Assessment, Total Maximum Daily Load Tracking and Implementation System (ATTAINS) regulatory database, which contains information on Section 303(d), 305(b), and Total Maximum Daily Load (TMDL) programs (US EPA 2016). These monitoring programs are part of a regulatory framework that jurisdictions and EPA have established to comply with Federal Clean Water Act requirements. The ATTAINS database contains information on monitoring efforts by each jurisdiction, including a water body’s designated use (e.g., drinking water, fish consumption, or ecological life uses), the stressors that have been assessed (e.g., acidity, dissolved oxygen (DO), sediment, etc.), and their status in relation to those designated uses (e.g., cause, meeting criteria, or insufficient information). Although jurisdictions may use different monitoring designs and stressor identification methods (Griggs and Buchanan 2012), all data are submitted into ATTAINS and therefore provide the most spatially complete perspective of the major stressors affecting stream biological impairment in the region.

Data for the most recent reporting period available in the ATTAINS database were pulled for each jurisdiction (2016 for West Virginia, 2018 for all others), and data were clipped to include only those assessment units contained within the CB watershed. The dataset was then further filtered to include only the free-flowing assessment units (“River”, “Stream/Creek/River”, “Stream”, and “Ditch or Canal”), excluding lentic and coastal waters. We harmonized terms used in the database to facilitate comparison across the records from the seven jurisdictions and to align with the literature review meta-analysis. We harmonized stressor categories, designated life uses, and suspected sources reported in ATTAINs (Fanelli and Cashman 2022). Since the focus of this study was on biological impairment, only records regarding a designated ecological life use for each jurisdiction were retained (e.g., aquatic life support, trout waters, warm water fishery). Finally, only records for which stressors were denoted as “Not meeting criteria” and a status identified as “Cause” were retained for analysis (Fanelli and Cashman 2022). To summarize the impairment information, stressor categories were based on the percentage of impaired stream miles within each jurisdiction and for the entire CB watershed. The reported suspected source of the impairment was also examined and summarized by percent stream miles for both individual jurisdictions and the entire CB watershed.

## Results

### Literature Review Results

**Fig. 2:**
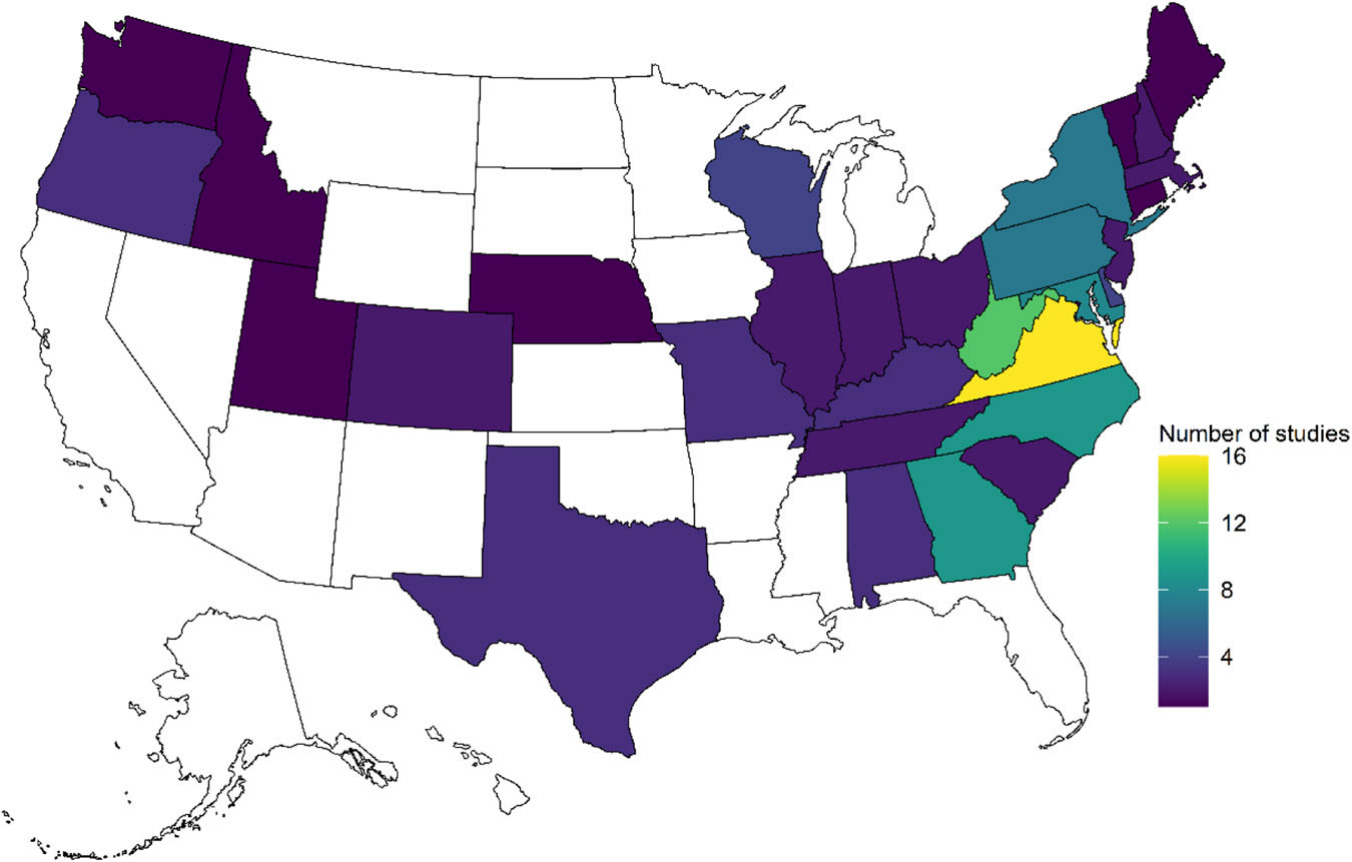
Number of multi-stressor studies identified in the literature review whose geographic extent included each state. Total number of studies in the United States = 65. Additional studies were conducted in Canada (6), New Zealand (3), Austria (1), Spain (1), Portugal (1), and England (1; see Fanelli and Cashman 2022 for details)

**Fig. 3:**
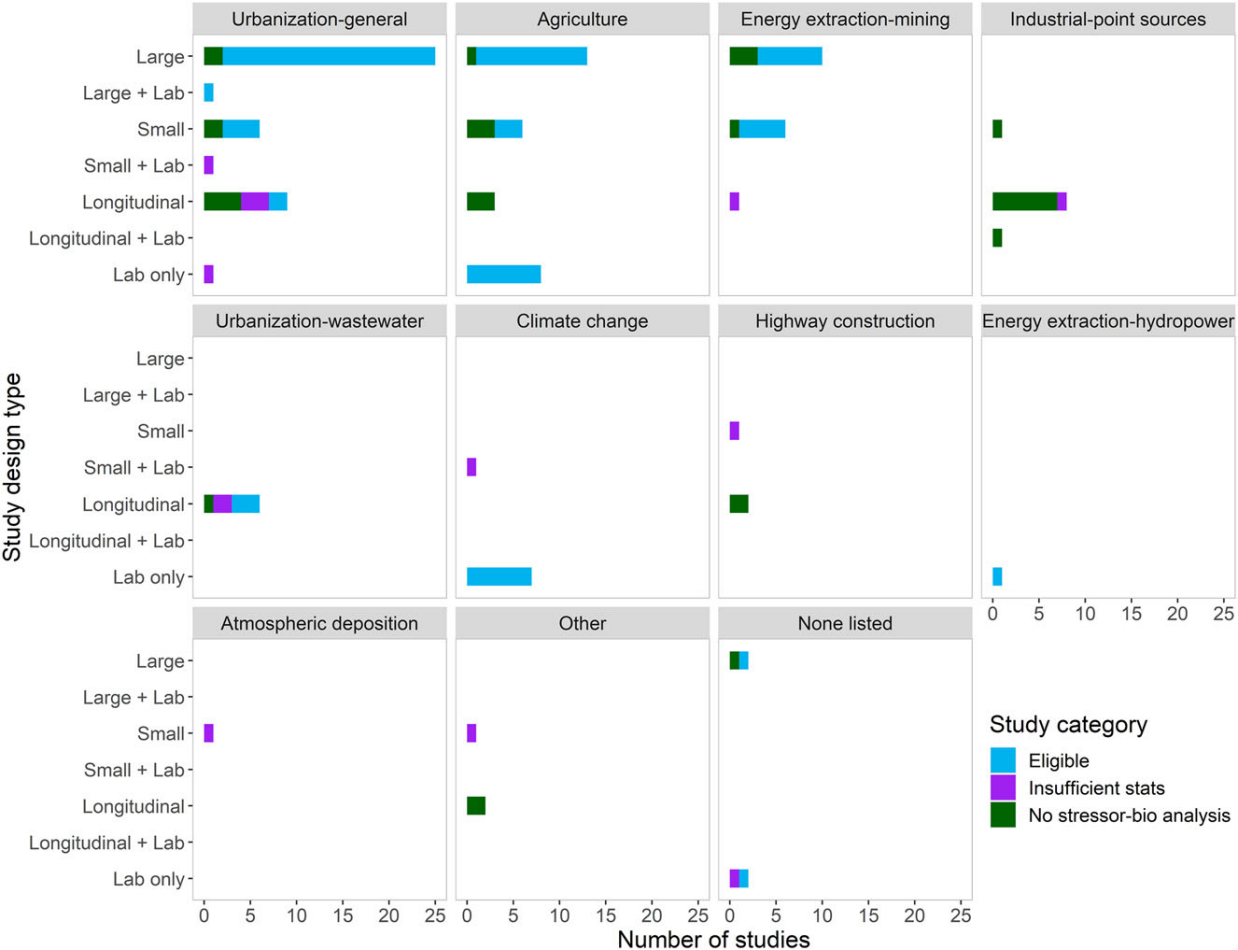
Number of studies (*x*-axis) reviewed for a given study design (*y*-axis) and suspected source (urbanization, point sources, etc.). Colors denote the status of the study in relation to the meta-analysis (blue = eligible; purple = not eligible due to insufficient statistics; green = not eligible due to the lack of explicit analysis between stressors and biological responses). See text for study design definitions. Some studies included multiple study designs (for example, a study may have reported on findings from field sites as well as a flume study, which is reported here as “Small + Lab”)

The initial literature search resulted in 78 multi-stressor studies mostly located in the Eastern United States (Fig. 2). Fourteen studies were eliminated because they did not meet initial eligibility criteria (for example, they examined relationships between landscape metrics and benthic communities; see Supplemental Information for details). The remaining 64 multi-stressor studies were considered for inclusion in the literature review and meta-analysis. Approximately 39% (25 studies) were large studies with 15 or more OUs, 27% (17) were longitudinal studies, and 23% (15) had a small study design with fewer than 15 OUs. The remaining seven studies were lab-based using flumes or mesocosms as OUs (note some studies may have employed multiple study designs). These multi-stressor studies spanned a range of sources, including urbanization (27 studies), agriculture (17 studies), mining (11 studies), industrial point sources (9 studies), and wastewater (5 studies; note studies may have focused on multiple sources; Fig. 3).

Of the 64 studies, about 52% (33) were eligible for the meta-analysis (see Fanelli and Cashman 2022 for a complete list). Twenty-one multi-stressor studies were considered ineligible because they did not explicitly relate biological response metrics to stressor data; these studies were often longitudinal studies which described biological and stressor patterns as a function of distance from the point source (e.g., Soucek et al. 2000; Echols et al. 2009; Fig. 3). Another ten studies did relate stressor data to biological response variables, but the statistical information provided in the studies was not aligned with our criteria for use to include in our meta-analysis. In the 33 studies that were included in the meta-analysis, a variety of benthic macroinvertebrate metrics were reported to reflect biological responses (Table 2). The most common ones we retained for our analysis were sensitive taxa richness (e.g., *Ephemeroptera*, *Plecoptera*, and *Trichoptera*, or EPT richness), analyses using measures of community dissimilarity (i.e., nonmetric multidimensional scaling or canonical correspondence analysis), and multi-metric indices (i.e., benthic index of biological integrity, or IBI; Table 2). The 33 eligible studies were mostly large studies leveraging spatial gradients of either agricultural land use or urban land use (mean of 62 OUs, median of 29 OUs, minimum of 5 OUs, maximum of 773 OUs; Fig. 3). The meta-analysis results below are reported for all eligible studies (*n* = 33), for agricultural studies only (*n* = 12) and for urban studies only (*n* = 18).

**Table 2:** Response metrics reported in the peer-reviewed literature to characterize benthic macroinvertebrate community conditions

Metric	Short name	Number of studies in meta-analysis reporting metric	Number of studies represented by metric in analysis
EPT richness	EPTRICH	15	14
Dissimilarity-based metrics	DISSIM	12	7
Multi-metric index	MULTI	11	5
Drift rates	DRIFT	4	3
Diversity	DIVERSE	4	1
Observed vs. expected	OE	4	1
Percent EPT taxa	PEREPT	1	1
Taxa richness	TAXARICH	10	1
Density	DEN	2	0
EPT abundance	EPTABUND	2	0
Evenness	EVEN	1	0
Other	OTH	1	0
Overall abundance	ABUND	1	0

Note: Some studies reported results for multiple response metrics; only one was chosen to avoid double-counting studies. *EPT* Ephemeroptera Plecoptera and Trichoptera (sensitive taxa)

#### All eligible studies

**Fig. 4:**
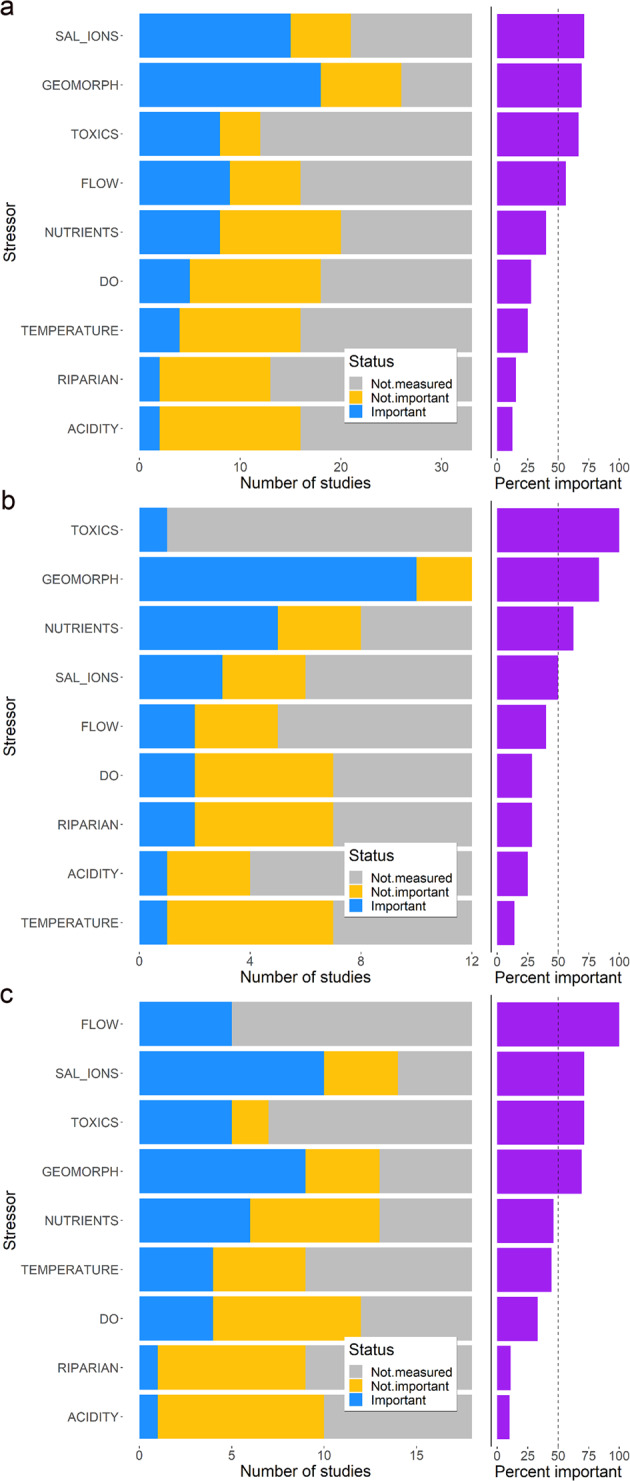
Barplot showing number of studies (*x*-axis) that measured a stressor category and reported it as important for explaining biological responses (left), and the percent of studies which measured the stressor also reporting it as important is shown on the (right) for (**a**) all studies (*n* = 33); (**b**) agricultural studies only (*n* = 12); and (**c**) urban studies only (*n* = 18). Vertical dashed line on right denotes 50%

Salinity or major ions, geomorphology, and toxic contaminants were most often reported as important for explaining patterns in biological responses (71%, 69%, and 62% of the studies measuring the stressor categories, respectively; Fig. 4a). Salinity and geomorphology were also among the most measured stressor categories, with 64% and 79% of the 33 eligible studies measuring them, respectively. Salinity, often reported as conductivity or specific conductance (SC), was found to be important mostly in urban and mining settings, whereas geomorphology—commonly reported as measures of in-stream habitat (Table 1)—was identified as a primary stressor across multiple land use settings (urban, agriculture, and mining). Toxic contaminants were measured in only 12 of the 33 studies, but 67% of those studies identified toxic contaminants as important (Fig. 4a). Toxic contaminants were linked to many different sources, including urbanization (pesticides, organic compounds, and metals), industrial or municipal wastewater (organic compounds), mining settings (metals), and agriculture (pesticides). Three of the four studies that measured pesticides (75%) found it to be important for explaining patterns in biological responses (Schmidt et al. 2019; Waite et al. 2019; Moran et al. 2020; Fig. SI-1). Similarly, three of the four studies that measured other toxic organic compounds, such as polycyclic aromatic hydrocarbons (PAHs), found them to be important (Bryant et al. 2012; Slye et al. 2011; Moran et al. 2020). By contrast, only four out of the nine (44%) studies that measured metals identified them as important for explaining biological responses (Fig. SI-1).

Flow was reported as important in more than half of the studies that measured them. Of the 16 studies that included some type of flow metric, nine (56%) found it was important for explaining patterns in biological responses. These studies were primarily focused on the effects of urbanization or hydropower energy generation. Although nutrients were commonly measured, only about 40% (8 out of 20 studies) reported nutrients as a primary stressor. Dissolved oxygen, stream temperature, riparian alteration, and acidity were rarely reported as important for explaining patterns in biological responses (28%, 25%, 15%, and 13% of the studies that measured them, respectively).

#### Studies in agricultural settings

Toxic contaminants, geomorphology, and nutrients had the highest percent reporting rates for explaining patterns in biological responses in agricultural settings (Fig. 4b). Although toxic contaminants had the highest percent reporting rates (100%), there is uncertainty in that value, given that only one agricultural study measured a toxic contaminant group (pesticides; Schmidt et al. 2019; Fig. SI-2). However, this study was among the largest included in our meta-analysis, for which 98 streams were sampled across a five-state region in the Midwest United States. Measures of geomorphology were included in all 12 studies, and 10 of them (83%) reported it as important. Fine sediment deposition was the most common geomorphic metric significantly associated with biological responses (Waite et al. 2014; Blocher et al. 2020; Piggott et al. 2012; Braccia 2006; Table 1). Other important metrics included riffle quality scores (Johnson and Ringler 2014; Maloney et al. 2011), and measures of streambed substrate composition or variability in streambed substrate composition (Braccia 2006; Roy et al. 2003; Johnson and Ringler 2014). Nutrients were important for explaining biological responses in 63% of the studies that included them. However, nutrients were often identified as indirect factors influencing other stressors such as DO or food web dynamics (Waite et al. 2019; Zheng et al. 2008; Barnes 2004). Most studies reported negative associations between biological response metrics and increasing nutrient concentrations. Total nitrogen, for example, was reported as one of the top five stressors explaining patterns in three different benthic community metrics (EPT taxa richness, tolerant taxa richness, and observed/expected) across 54 agricultural watersheds in the Eastern United States (Waite et al. 2014).

Salinity and major ions and flow were reported to be important in about 40–50% of studies in which they were reported (Fig. 4b). The role of salinity as a stressor in agricultural settings remains unclear since the study area in two of the three studies measuring salinity spanned both urban and agricultural gradients (Johnson and Ringler 2014; Roy et al. 2003). The third study was conducted in a carbonate setting, further confounding the effects of agriculture on salinity. Flow was important in two of the five studies, including one that used flumes to investigate the effects of water abstraction for irrigation, as well as the interactive effects of nutrient enrichment and fine sediment additions (Matthaei et al. 2010). Dissolved oxygen, riparian conditions, acidity, and stream temperature were rarely reported (<30%) as important for explaining patterns in biological responses in agricultural settings.

#### Studies in urban settings

Flow, salinity or major ions, toxic contaminants, and geomorphology had the highest percent reporting rates for explaining patterns in biological responses in urban settings (Fig. 4c). Flow was rarely measured (5 of 18 studies – 28%) but was always an important factor for explaining biological responses when measured. Metrics describing hydrologic alteration were identified as affecting benthic community metrics. For example, high-frequency stage and rainfall data were used to quantify the frequency of runoff events in 11 stream reaches along an urbanization gradient (Fanelli et al. 2019). In this study, urban sites had greater runoff frequency and lower EPT taxa richness. Peak flow interval was another flow metric used to describe hydrologic disturbances and was among the top five stressors explaining patterns in EPT taxa richness and total taxa richness in 76 streams across an urbanization gradient in the southeastern United States (Waite et al. 2019).

Salinity or major ions were important stressors in 10 of the 14 studies that measured them (71%) and were mostly expressed as discrete measures of SC (i.e., Roy et al. 2003a; Walters et al. 2009), or ion concentration (i.e., Liao et al. 2018; Bazinet et al. 2010). For example, Roy et al. 2003a examined water quality, physical habitat, and land cover characteristics in 30 sites spanning an urbanization gradient and used these metrics in a multiple regression analysis to explain variability in a suite of benthic invertebrate metrics. Specific conductance was included as a top predictor in models explaining variability in total taxa richness, EPT taxa richness, and benthic IBIs.

Toxic contaminants were important stressors in five of the seven (71%) urban studies that measured them (Fig. 4c), which included hydrophobic organic contaminant concentrations in water (Bryant and Carlisle 2012), pesticide concentrations in water (Waite et al. 2019; Moran et al. 2020; Schmidt et al. 2019), organic contaminants in streambed sediments (Waite et al. 2019; Moran et al. 2020), and surfactant water concentrations (Slye et al. 2011; Fig. SI-3). For example, a multi-state study examined stressors and macroinvertebrate communities at 76 sites spanning an urbanization gradient in the southeastern United States (Waite et al. 2019). Boosted regression trees identified multiple toxic contaminants driving three macroinvertebrate community metrics (EPT richness, taxa richness and observed vs expected, or O/E), including insecticides and their degradates, a multi-metric indicator of sediment contamination, and the median number of pesticides detected (Waite et al. 2019).

Geomorphology was important in 9 of the 13 studies (69%) that measured it. Geomorphology was expressed mostly by qualitative scores from rapid habitat assessments (Table 1). For example, cobble substrate scores and percent riffle cover were among the top three measures explaining variability in EPT taxa richness across 17 sites with variable urban land use in upstate New York (Johnson et al. 2014). Some studies conducted quantitative habitat assessments as well— a study of 30 stream reaches spanning an urbanization gradient in the Atlanta, GA region found streambed substrate variability and mean water depth were important predictors of biological response variables, both of which were quantified by detailed geomorphic surveys (Roy et al. 2003a). Nutrients and stream temperature were identified in slightly less than than half of the urban studies as important for driving biological response patterns (Fig. 4c). Dissolved oxygen, riparian alteration, and acidity were rarely identified (<30%) as the primary stressors driving biological responses in urban streams.

### Jurisdictional Impairment Analysis Results

**Fig. 5:**
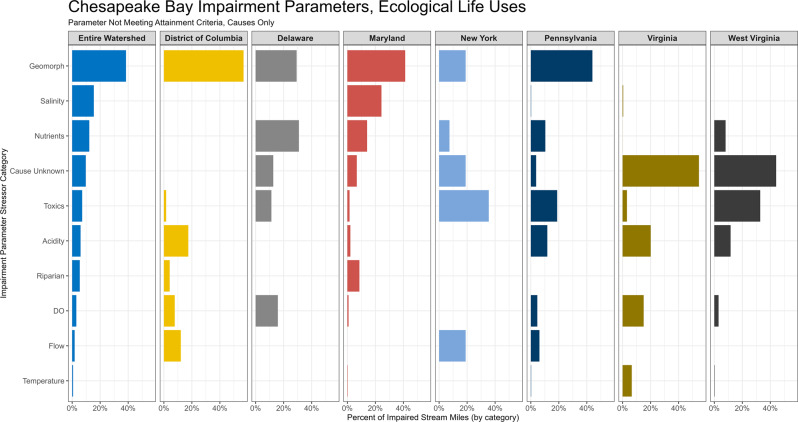
Percent of all impaired stream miles causally attributed to a stressor category within each jurisdiction. Percent is of the sum of stream miles of all impairments within a jurisdiction and stream reaches in some jurisdictions may be counted multiple times if covered by multiple impairments. Dissolved oxygen (DO)

**Fig. 6:**
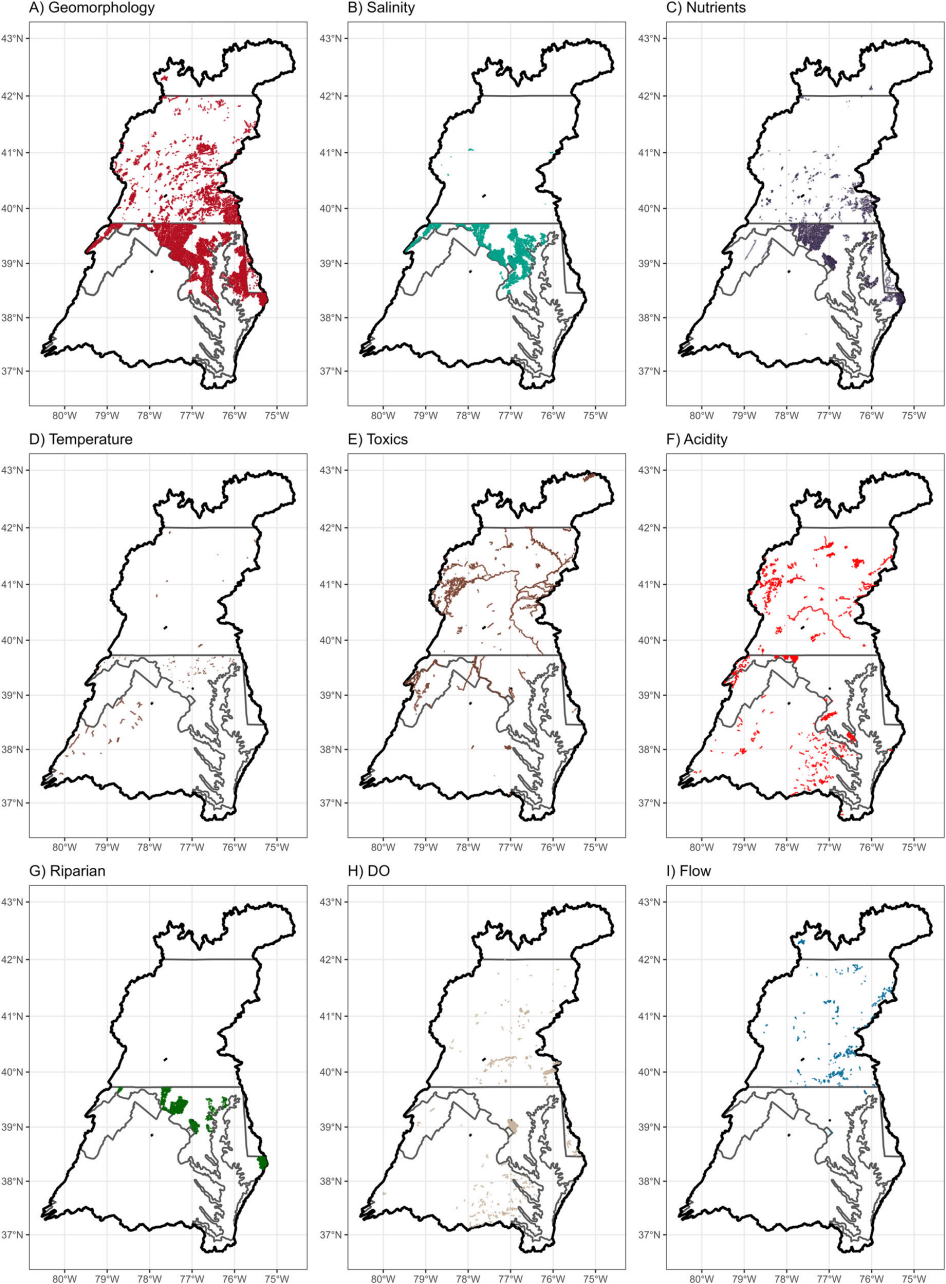
Spatial distribution of jurisdiction-reported impairments caused by the nine major stressor categories. From left to right: (top row) geomorphology, salinity, nutrients; (middle row) temperature, toxics, acidity; (bottom row) riparian condition, dissolved oxygen, flow

Across the entire CB watershed, the most reported stressors for impairing ecological life uses were geomorphology, salinity and major ions, and nutrients (Fig. 5), broadly aligning with the general findings in the literature review meta-analysis (Fig. 4a). Top stressors, however, varied greatly among jurisdictions and likely reflect differences among the stressor identification methodologies each jurisdiction employs for their assessment programs, as well as differing regulatory focus, philosophy, and policies (Figs. 5, 6). For example, geomorphology was listed as the predominant stressor in the majority of stream reaches in four jurisdictions (DC, MD, PA, and DE), but not listed as a stressor at all in three others (VA, WV, and NY). Within the geomorphology category, jurisdictions predominantly reported either sediment (DC and MD) or physical habitat (DE, NY, and PA; Figure SI-4) as the primary cause of impairment. Salinity ranked second in terms of stream miles, but almost all these reaches were located in just one jurisdiction (MD). Nutrients were reported in five of the seven jurisdictions, but were among the top three stressors reported in only DE and MD (Fig. 5).

Toxic contaminants, acidity, and riparian conditions were sometimes reported as the primary stressor (Fig. 5). Toxic contaminants were moderately prevalent across the entire region and were among the top three stressors reported in NY, PA, and WV. Mercury was the only toxic contaminant group reported in NY (Fig. SI-4). Metals were the most reported toxic contaminant class overall, and the only toxic contaminant class reported by WV. Pennsylvania reported reaches to be impaired by mercury, non-pesticide organic contaminants, or metals. Pesticides were rarely reported in the region as the cause of biological impairment (Fig. SI-4) and those that were reported were from exclusively legacy and banned pesticide types. Acidity issues were reported in five of the seven jurisdictions and were among the top three stressors in DC, PA, VA, and WV. Riparian alteration was never listed as a top stressor in any jurisdiction.

Dissolved oxygen, flow, and stream temperature were rarely reported across the entire region as a primary stressor (Fig. 5). Dissolved oxygen was reported in six of the seven jurisdictions (all but NY) but only reported among the top three stressors in DE and VA. Flow was reported in three of the seven jurisdictions, but commonly was used to indicate effects of flow regulation, like hydropower, and was among the top three stressors reported only in NY. Stream temperature was reported in four jurisdictions (MD, PA, VA, and WV), with Virginia reporting it the most frequently as an ecological stressor. There was also a “cause unknown” category frequently reported by multiple jurisdictions (DE, NY, VA, and WV; Fig. 5). Typically, an observed impairment of the biological community will trigger a stressor identification assessment to identify the stressor causing that impairment. Once the stressor has been identified, the database will be updated with the new stressor cause information. However, as of this study, much of the ecological impairment information for VA and WV have not yet been updated after the identification process within the ATTAINs database, despite identified stressors being included in state-level integrated reports and regulated within individual TMDLs. In addition, it is worth noting that many streams in VA and WV are reported as impaired by pathogens for drinking water and recreational uses (Fig. SI-5); these listings were excluded from the analysis since the focus of this study was on ecological uses only.

**Fig. 7:**
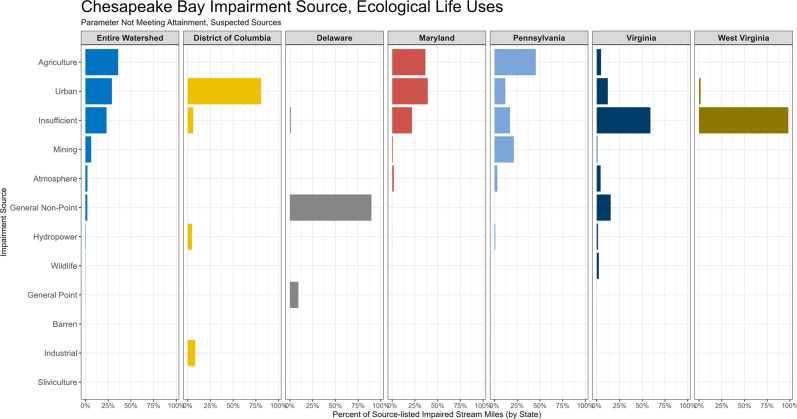
Percent of impaired stream miles that have a listed suspected source of impairment for each jurisdiction. New York is not included since no suspected sources were reported

Jurisdictions reported agricultural and urban land uses frequently as the suspected source of impairment (Fig. 7). Agricultural land use was only largely reported as a source in MD and PA and never specifically reported in three jurisdictions (DE, WV, and DC). Urban land use was reported as the source in six of the seven jurisdictions, and most frequently reported in DC and MD. Mining activities were reported to be somewhat prevalent in PA and to a lesser extent in VA and MD. All jurisdictions, and especially VA and WV, reported potential source information for which there was insufficient information to designate a specific source (e.g., from upstream, tidal, etc.; Fanelli and Cashman 2022). Delaware used a different approach to report suspected sources of biological impairment — rather than use land use categories as sources, general point and general non-point sources were reported, with general non-point sources as the primary source of impairment. Industrial sources were only reported in DC, and hydropower was reported infrequently in DC, PA, and VA. Atmospheric deposition was reported as a suspected cause in MD, PA, and VA.

## Discussion

The two analyses conducted in this study provided complementary perspectives for understanding key stressors driving freshwater stream biological impairment in the CB watershed. There were multiple points of agreement between the two analyses, but also notable differences among different landscape settings, sources of impairment, and among the seven jurisdictions, which has implications for allocating resources and selecting strategies for resource management, conservation, and restoration. In the discussion, we 1) compare and synthesize results from the two analyses and discuss linkages between stressor categories; 2) briefly discuss findings from the literature review on other measures of stream health (Fig. 1a); 3) address potential sources of impairment that were not sufficiently addressed in the analysis; 4) discuss the limitations of each data source and analysis; and 5) articulate lessons learned and future research directions identified through this study.

### Comparison of the Literature Review and the Jurisdictional Impairment Analysis

Four elements from the analysis were considered for this comparison: how frequently a stressor was reported as important in the meta-analysis; the frequency of the stressor being measured in the literature review; and how prevalent the stressor was reported in the jurisdictional impairment analysis at two scales: across the region overall, and within individual jurisdictions (Table 3). We review all findings and discuss general conclusions for the nine major stressor categories below.

**Table 3:** Synthesis of findings from the literature review and jurisdictional impairment analysis by major stressor category. Linkages to other stressors reflect the network diagram shown in Fig. 1b

Stressor category	Literature review		Jurisdictional impairment analysis		Linkages to other stressors		General conclusions
	Frequency of being reported as important	Frequency of measurement	Watershed-wide ranking	Jurisdictions in which stressor is among top three reported	Can be affected by	Can affect	
Geomorphology	High	High	High	DC, DE, MD, PA	Flow, riparian alteration	Toxic contaminants, nutrients, dissolved oxygen, stream temperature	Most likely a primary stressor throughout region, especially in agricultural settings
Salinity and major ions	High	High	High	MD	Toxic contaminants (e.g., metals)	Nutrients, toxic contaminants (e.g., metals)	Likely a primary stressor in urban, mining, and point-source settings; Needs more study in other jurisdictions
Nutrients	Moderate	High	High	DE, MD	Geomorphology, flow, riparian alteration	Dissolved oxygen	Likely a contributing stressor, and could also act as a proxy for another co-occurring stressor (e.g., DO)
Toxic contaminants	High	Low	Moderate	NY, PA, WV	---	Salinity and major ions	Likely a primary stressor where sources exist; needs more study
Flow	Moderate	Low	Moderate	DC, NY	---	Geomorphology, toxic contaminants, nutrients, dissolved oxygen, stream temperature	Likely a primary or contributing stressor where the hydrologic cycle has been altered, needs more study
Acidity	Low	Moderate	Moderate	DC, PA, VA, WV	---	Toxic contaminants (e.g., metals)	Not likely a contemporary primary stressor except in regions with historic mining operations or high atmospheric deposition
Riparian	Low	Low	Moderate	none	---	Stream temperature, geomorphology, nutrients	Could be a contributing stressor, especially in agricultural regions
Dissolved oxygen	Moderate	Moderate	Low	DE, VA	Nutrients, flow, geomorphology, stream temperature	---	Could be a primary stressor in reaches with high nutrients/biological oxygen demand, low aeration rates, or fine sediment deposition are present
Temperature	Low	Moderate	Low	none	Riparian conditions, geomorphology, flow	Dissolved oxygen	Likely a primary stressor in cold-water stream reaches where no other stressors are present

The terms “High”, “Moderate”, or “Low” denote the stressor’s overall rankings in Figs. 4A and 5 (“High” = ranks 1–3; “Moderate = ranks 4–6; “Low” = ranks 7–9). Data sources are included in Fanelli and Cashman (2022)

*DC* District of Columbia, *DE* Delaware, *MD* Maryland, *PA* Pennsylvania, *NY* New York, *VA* Virginia, *WV* West Virginia

#### Geomorphology

*Both analyses indicate altered geomorphology (physical habitat and/or excess sediment loading) as a likely regional primary stressor affecting biological communities, especially in agricultural settings*. Geomorphology was the most common measured stressor category and had the second-highest percent importance ranking (Fig. 4a). Sediment is a known issue within the CB watershed and has been identified as one of the top pollutants of concern for CB health (U.S. EPA 2010; Noe et al. 2020). While erosion from land use such as agriculture is a major factor across the region, a substantial fraction of in-stream excess sediment originates from in-channel erosion (Gellis, Gorman Sanisaca 2018), and most dramatically in urban areas (Cashman et al. 2018). While elevated in-channel erosion can occur solely due to historic disturbances decades or centuries in the past (e.g., legacy sediment, James 2013; Lyons et al. 2015), in-channel erosion and instability are notably caused by another stressor – altered flow regimes – due to increased stormwater runoff in urban areas (Booth 2005).

Excess sediment’s primary effect on macroinvertebrates is through deposition and siltation of the bed and degrading local bed habitat conditions, which is captured by metrics such as embeddedness (Jones et al. 2012). Fine sediment can be a vector for toxic contaminants or nutrients, and high rates of fine sediment deposition can decrease oxygenation rates in interstitial spaces in streambed sediments and hyporheic zones, potentially triggering hypoxia in those settings. Other geomorphic disturbances such as over widening, ditching, straightening, incision, or other forms of channel homogenization can reduce the channel’s resiliency to excess sediment by impairing geomorphic processes such as sediment sorting by particle size or floodplain deposition. As a result, degraded bed conditions can occur due to these other geomorphic alterations without explicitly altering sediment loads. Therefore, management strategies could focus on restoring habitat at scales relevant to biota and address both in-channel and upstream watershed factors that control this stressor, such as flow, as well as prioritizing the restoration of geomorphic function—not just forms—which can assist with channel resiliency to excess sediment pressure (Friberg et al. 2016). Consideration of the effects of altered geomorphology and sediment on other stressor categories as well (Fig. 1b) is important to fully understand its impacts on stream ecosystems.

#### Salinity and major ions

*This stressor category is likely a primary or contributing stressor in urban, industrial, and mining settings in the region*. Salinity and major ions were very often measured and reported as important in the literature review meta-analysis. More research is needed, however, to determine how pervasive this stressor category is across the region since it was only heavily reported in MD in the jurisdictional impairment analysis (Fig. 6, Table 3). In urban settings, deicer and anti-icing material, such as NaCl or MgCl either in granular or liquid/brine form, is likely a major source of elevated ions (Corsi et al. 2015), though other sources include weathering of impervious surfaces (Kaushal et al. 2018). Urban soils, groundwater, or even stormwater management ponds also may be sources of excess ions (Snodgrass et al. 2017). In mining settings, increased ionic strength in streams originates from increased weathering of excavated material (Griffith et al. 2012), and mixtures of multiple ions released from surface coal mining likely causes biological impairment (Cormier et al. 2013). Other types of energy extraction activities, such as oil and gas extraction or deep mining, generate produced waters, often high in SC, which could discharge to streams or groundwater (Engle et al. 2014). Industrial sites may also discharge brines into freshwater streams and raise salinity downstream of the outfall (Echols et al. 2009).

Most studies reported SC rather than a specific ion as the primary stressor since SC is much easier and less expensive to measure. However, SC can be a proxy for ions or contaminants, such as bicarbonate, chloride, or metals, that are themselves stressors (Cormier et al. 2013; Moore et al. 2020; Galella et al. 2021). Benchmark concentrations of ions above which aquatic organisms are intolerant may depend on other factors, such as temperature (Jackson and Funk 2019) or concentrations of other ions in the mixture (Scheibener et al. 2017). High-frequency (i.e., sub-daily) SC data can reveal highly dynamic patterns that are often missed by infrequent discrete sampling campaigns (Moore et al. 2020). When coupled with discrete measures of major ions, high-frequency SC may help characterize sources and transport of ions in streams. The long-term consequences of the activities that lead to elevated salinity could be considered by managers. For example, water quality issues may persist in streams draining mining operations long after operations have ceased (Pond et al. 2014). Long-term and repeated application of deicer material may also accumulate in urban soil and groundwater sources, causing stream salinity to remain elevated long after application rates have declined or ceased. Deicer applications can also affect soil cation exchange processes, including the release of other constituents, such as metals or nutrients (Duan and Kaushal 2015).

#### Nutrients

*Nutrients are likely a contributing stressor to biological impairment but could also be serving as a proxy or co-occurring stressor*. Nutrients were often measured but were reported to be important for explaining biological conditions in less than half of those studies in the literature review (Fig. 4a). However, nutrients were often listed as the cause of biological impairment by MD and DE in the jurisdictional impairment analysis. Nutrients contribute to biological impairment indirectly, whereby elevated nutrients cause decreases in DO levels and shifts to more tolerant taxa (Zheng et al. 2008; Yuan and Norton 2003). Excess nutrients decrease nutritional quality of food sources for benthic macroinvertebrates as well (Cashman et al. 2013). Algal blooms associated with eutrophication from excess nutrient inputs can also cause pH to rise above neutral conditions, but it is unclear how prevalent this factor is in the region. Although DO was measured in some studies, the studies were often based on limited sampling and may not reflect dynamic conditions that organisms experience throughout their life stages (see DO section below). A recent national-scale synthesis of stressor studies found the negative effects of nutrients were outweighed by other stressor categories, like toxic contaminants, for predicting benthic community metrics (Waite et al. 2021), suggesting they may not necessarily be the primary cause of ecological impairment in local waterways. Elevated nutrients are common in the region due to widespread agricultural activities, but could co-occur with other stressors associated with agriculture, like pesticides, which are rarely measured. The frequency of nutrients listed as the primary cause of biological impairment in the jurisdictional impairment database may reflect the regularity and ease of measurement by which many municipalities monitor nutrient levels in local waterways to adhere to regional TMDL monitoring programs. It is therefore important to consider other co-occurring or proxy stressors that could be influential factors affecting biological impairment.

#### Toxic contaminants

*Toxic contaminants are likely primary stressors where sources exist, but more monitoring is needed to better understand the extent and severity of contaminants across the region*. Although rarely measured, toxic contaminants, and especially pesticides, were important across multiple land use settings in the literature review meta-analysis. Pesticides and other organic contaminants are increasingly being identified as a key stressor shaping benthic communities across the United States (Waite et al. 2021) and in Europe (Liess et al. 2021; Beketov et al. 2013). A nation-wide study recently found agricultural pesticide levels in 74 rivers across the United States above ecologically relevant thresholds (Stackpoole et al. 2021). Pesticides have also been consistently reported as a primary stressor or above toxicity levels in urban centers across the United States (Brown et al. 2009; Waite et al. 2019; Nowell et al. 2021). By contrast, pesticides and other organic contaminants were rarely reported in the jurisdictional impairment analysis, and the only pesticide impairments were for legacy banned substances, suggesting these contemporary toxic contaminants are likely understudied across the region. Metals were the most reported toxic contaminant stressor by jurisdictions (Fig. SI-4), which may be highlighting the prevalence of legacy mining activities in the region. Indeed, jurisdictions that have a strong mining history (PA and WV) reported metals most frequently in the jurisdictional impairment analysis. Heavy metals can also originate from urban settings (Ferreira et al. 2016). Although metals could still be a chronic issue in mining settings, more work needs to be done to evaluate the extent and severity of metal contamination across the region. Little research was found that focused on other toxic contaminants, like polychlorinated biphenyls (PCBs), pharmaceuticals, and endocrine disrupting compounds in freshwater streams, and more work is needed to understand the effects of these contaminants on freshwater stream health (Waite et al. 2019; Moran et al. 2020).

#### Flow

*Flow regime alteration is likely a primary or contributing stressor where the hydrologic cycle has been altered (e.g., in reaches with hydropower operations or in urban streams with increased stormwater runoff)*. Though rarely measured, flow was found to be important for explaining biological responses in the literature review, especially in urban settings. By contrast, flow was rarely reported in the jurisdictional impairment analysis; only three jurisdictions reported flow as a primary stressor (DC, NY, PA). Flow is often considered the “master variable” controlling the occurrence and distribution of aquatic organisms in streams and rivers (Poff et al. 1997), and many different anthropogenic activities alter flow patterns in downstream reaches. Urbanization, for example, has been linked to higher peak flow magnitude and frequency of high flows (Walsh et al. 2012; Hopkins et al. 2020), and hydropower dam operations reduce hydrologic variability, which is critical for supporting life stages of aquatic organisms (Poff et al. 2006; Bunn, Arthington 2002). Other activities, such as irrigation for agriculture or groundwater withdrawals for water supplies, may alter flow regimes in nearby reaches by depleting groundwater sources. Flow regimes are tightly coupled to watershed sediment dynamics, channel stability, and physical habitat conditions (Booth and Jackson 1997), highlighting its role as a contributing factor when geomorphology and sediment is identified as a stressor. In such cases, management actions are unlikely to improve bed habitat conditions without addressing flow modification, even if landscape erosion from the upslope land uses are reduced. Consideration of flow alteration as a potential stressor is warranted in these settings. Flow regimes can also have indirect effects on many other stressor categories, including DO, nutrient and contaminant transport, and stream thermal regimes, further highlighting the important control flow regime has on overall stream health.

#### Acidity

*Acidity is not likely a primary stressor except in limited areas with legacy mining contamination or in forested streams with elevated atmospheric deposition rates*. Although acidity was rarely reported as an important stressor for benthic community in the meta-analysis, it was among the top three reported stressors in four jurisdictions (DC, PA, VA, WV). One of the sources of low pH in these regions is acid mine drainage from legacy coal mining operations (Chesapeake Bay Program 1998). Benthic diversity and abundance often decline in streams impacted by acid mine drainage (Hogsdon and Harding 2012). Low pH can cause physiological stress and prompt drifting behavior in benthic macroinvertebrates (Courtney and Clements 1998) and can affect the bioavailability/redox condition of metals, such as aluminum, which may also impact biota (Mulholland et al. 1992). Another documented source of low pH in the region is atmospheric deposition (Jastram et al. 2013). Both legacy acid mine drainage and atmospheric deposition may continue to be key sources of biological impairment in forested stream ecosystems in the future (Bott et al. 2012; Rice et al. 2014).

#### Riparian alteration

*Degraded riparian conditions could be a contributing stressor, especially in agricultural regions*. Riparian alteration was rarely identified as a stressor in the meta-analysis and was not among the top three stressors in any jurisdiction, indicating limited evidence of it being a primary stressor. However, benthic invertebrate metrics are often positively associated with increased riparian cover (Rios, Bailey 2006), suggesting a loss of riparian cover may negatively impact stream ecosystems. Riparian conditions can have indirect effects on stream health metrics by enhancing stream resiliency and mitigating the effects of other stressor categories, including stream temperature (Dan Moore et al. 2005), and intercepting overland flow and delivery of nutrients and sediment. Moreover, vegetated riparian zones control the type and abundance of detrital material entering the stream channel, which serves as energy resources of omnivorous aquatic organisms (Braccia, Voshell 2006). Riparian vegetative cover also regulates light availability, which in turn affects algal growth in the channel (Sabater et al. 2000) and can alter the nutritional quality of food resources for consumers (Cashman et al. 2013). Managers may consider riparian zone enhancement and protection as a viable strategy to mitigate the effects of agriculture on multiple in-stream stressors.

#### Dissolved oxygen

*Dissolved oxygen could be a primary stressor in reaches with high nutrients/biological oxygen demand, low aeration rates, or fine sediment deposition*. High oxygen demand often occurs in reaches with high levels of organic matter inputs and nutrient concentrations. This can occur in agricultural settings (dos Reis Oliveira et al. 2019) or downstream from wastewater treatment plants (Ortiz and Puig 2007). Low DO can also occur in urban streams, where low aeration rates from altered geomorphology occur (e.g., stagnant pools; Blaszczak et al. 2019). Dissolved oxygen was rarely reported as important in the literature review meta-analysis. This could be due to the presence of other stressors, like nutrients, being more strongly associated with biological response metrics. Alternatively, DO dynamics may not have been adequately characterized in these studies, given its strong diel pattern – many studies relied on discrete measures to characterize DO conditions (Table 1). Dissolved oxygen was identified as one of the top stressors in a large, regional urbanization study in the southeastern United States (Waite et al. 2019) and was attributed to high nutrient concentrations in those streams. The stream reaches in the CB watershed that were reported in the jurisdictional impairment analysis as impaired by nutrients are likely impaired by low DO conditions. Moreover, DO levels are affected by temperature, so factors that negatively affect stream temperature may exacerbate this stressor condition. Consideration of DO is warranted in reaches with elevated nutrient and organic matter inputs and/or indicators of altered thermal regimes (low riparian cover, extensive urban runoff inputs).

#### Temperature

*Stream temperature is likely a primary stressor in cold-water stream reaches where no other stressors are present, and a contributing stressor in urban streams or reaches that are susceptible to hypoxia*. Stream temperature was not regarded as a primary stressor in most studies in the meta-analysis, and no jurisdiction reported it as a top-ranked stressor (Table 3). This suggests either other stressors present exerted more pressure on aquatic organisms, or that stream temperature was poorly characterized in the stressor studies. For example, many of the studies that considered temperature used few discrete measures of water temperature (e.g., Frondorf 2001; Meador et al. 2008), which may not adequately characterize conditions in the stream. High-frequency temperature monitoring may better characterize changes in thermal regime (e.g., Fanelli et al. 2019) and provide a better understanding of how temperature impacts stream biological communities. In the absence of any other stressors (contaminants, flow alteration, etc.), rising stream temperatures due to climate change is a likely stressor for cold-water dependent benthic organisms (Richards et al. 2018; Rice and Jastram 2015) and may also cause shifts in benthic community composition in reference streams across the region in the future.

### Other Measures of Stream Health Reported in the Multi-stressor Literature

This analysis focused on stressors influencing patterns in commonly reported benthic community metrics (e.g., multi-metric IBIs or EPT taxa richness), which describe different elements of community structure. Stressor-response relationships often can be complex (i.e., non-linear) and often divergent for different measures of benthic community dynamics, and can differ across multiple stressor gradients (for example, habitat quality, ionic strength, phosphorus, and metals; Yuan et al. 2003). Reference-based metrics, such as differences in observed versus expected community composition (i.e. observed vs. expected, or O/E) or dissimilarity metrics, do reflect changes in communities as a whole relative to reference communities, but it may be difficult to identify mechanistic pathways from stressor to impairment. Other structural metrics, such as gill morphology, may better characterize the mechanism by which the stressor impairs organisms, as seen in mayfly larvae impacted by methylmercury contamination from atmospheric deposition (Skinner, Bennett 2007) or elevated ion concentrations (Orr et al. 2022). Trait-based metrics, which describe life history, physiological, and reproductive characteristics, may more clearly document how stressors affect ecosystem function (Nichols et al. 2016), since they may be more sensitive to specific stressors than other types of metrics (Poff et al. 2010). Secondary production measures of benthic communities may also reveal the impacts of stressors on food web dynamics (Johnson et al. 2013).

Other biological communities, such as fish or diatoms, may respond differently than benthic macroinvertebrate communities across the same multi-stressor gradient. For example, fish community metrics were more sensitive to variability in DO and stream temperature than benthic metrics in 94 sites spanning an urbanization gradient in the northeastern United States (Waite et al. 2021). Diatom metrics, on the other hand, were most sensitive to nutrient concentrations across the same gradient. In MD, habitat metrics, such as percent embeddedness, exerted greater control on explaining variability in fish community than benthic community metrics, while the reverse was true for water quality metrics, such as salinity and nutrients (Walker et al. 2021). Relationships between stressors and metrics describing fish health is also understudied, and efforts to determine stressors driving patterns in fish health metrics may help illuminate mechanistic pathways of biological impairment (Matsche et al. 2021). Diatom responses are also helpful for understanding benthic macroinvertebrate dynamics since they are a food resource for some benthic organisms. Incorporating multiple metrics of stream health may be necessary to fully understand the effects of anthropogenic disturbances, such as land use change, on stream ecosystems.

### Additional Sources of Impairment and their Key Stressors

Agricultural, urban, and mining settings were the most common sources of impairment examined in the literature review (Fig. 3) and reported in the jurisdictional impairment analysis (Fig. 7). Other sources of impairment, including point source discharges, other resource extraction activities, and climate change, also likely influence in-stream stressors and stream health in the CB watershed. Point sources, for example, can drastically alter water quality and contaminant loading in reaches immediately downstream from the discharge point, and includes industrial discharges (Hall et al. 2009; Nedeau et al. 2003; Echols et al. 2009; Monda et al. 1995) and municipal wastewater (Polls et al. 1980; Birge et al. 1989). Chemical mixtures containing multiple contaminants can originate from municipal or industrial wastewater discharges, and those mixtures can collectively increase ecotoxicological impacts to aquatic organisms (Barber et al. 2022). Impacts from point sources can occur in agricultural settings as well, including increased nutrient loading from aquaculture activities (Koetsier 2002) or slaughterhouse discharges (Roberts 2005). Power generation activities, such as coal combustion for energy generation, may also include point source discharge effluent high in metals, which can impact downstream biota (Reash 2004).

Oil and gas extraction activities, including unconventional oil and gas (UOG) operations which had expanded dramatically in the early 2000s in PA, could pose risks to aquatic ecosystems. Research on the biological effects of UOG extraction is limited, so it was not represented in the literature review meta-analysis. However, a recent study examined chemical tracers and biological communities in 25 reaches across a UOG intensity gradient and found little evidence of direct impacts on biota via chemical stressors or habitat changes from the UOG footprints (Mumford et al. 2020). Timber harvesting in forested regions of the CB watershed could potentially impact stream ecosystems via multiple pathways, including degraded riparian conditions and increased nutrient and fine sediment loading (Fortino et al. 2004). Altered rainfall patterns from climate change (Ning et al. 2015) will likely alter stream and river flow regimes, which influences benthic macroinvertebrate communities in the region (Maloney et al. 2021). Rising stream temperature from climate change in the region (Rice and Jastram 2015) may contribute to shifts in regional distributions of cold-sensitive species, such as brook trout (Snyder et al. 2015).

### Study Limitations

#### General limitations of stressor identification assessments

Our study relied on the results of two sets of stressor identification assessments (peer-reviewed literature and the jurisdictional impairment assessments), and differences in their study design and analysis could have introduced uncertainty in their findings. In all studies, prior knowledge about a possible source causing biological impairment could have influenced the stressor groups chosen to be monitored. For example, physical habitat may not often be included as a potential stressor in assessments focused on mining settings, since the impairment often is hypothesized to be driven solely by water quality (but see Drover et al. 2020; Pond 2010, and Chambers and Messinger 2001 as exceptions). Incomplete inclusion of all possible stressors may introduce greater uncertainty in the findings since a co-occurring or proxy stressor may be mis-identified as the most influential primary stressor.

Logistical, technical feasibility, or financial constraints may have limited the number of stressors measured in the peer-reviewed literature and in the jurisdictional stressor identification assessments. Stressor categories that are difficult and expensive to characterize, such as toxic contaminants and flow alteration, are quite understudied (Fig. 4). It is understandable to limit monitoring to only those stressors most likely to be present given study constraints, but there needs to be careful consideration of the risk of identifying a confounding stressor rather than the actual cause given the highly complex linkages among stressor categories (Fig. 1b). Another factor contributing to differences between results is how the stressor condition is characterized, which may also be influenced by cost and logistical limitations. Infrequent, discrete measures of water quality (e.g., single samples), for example, may not adequately reflect the range in conditions that organisms are experiencing throughout their life cycle.

The nature of the source (e.g., presumed to be either diffuse or point source), may also influence the study design. For example, point source studies often adopted longitudinal sampling in their assessments (e.g., Polls et al. 1980; Fig. 3), whereas studies focused on general urban or agricultural land use effects often relied on spatial gradient study designs. Study designs influence the types of statistical analysis that can be conducted on the data, and some analyses are more powerful for stressor identification than others (e.g., comparison of simple correlations vs. non-linear boosted regression trees). Moreover, other metrics of habitat suitability that reflect larger spatial scales (e.g., regional habitat connectivity) are not often quantified in stressor identification assessments but may also play a role in shaping fish community composition (Samia et al. 2015). Biotic factors, such as intra-species competition, also likely plays a role in biological response patterns but are not included in many studies.

Differences in assessment methodologies between jurisdictions were apparent in our study (Fig. 6). While comparisons of the regulatory data in the ATTAINS database provide a broad overview of patterns in the CB watershed by state, differences in the various methodologies, reporting styles, and reported details across jurisdictions resulted in substantial differences in patterns of stressor prevalence, despite our efforts to harmonize results across the region. This variability may be a source of significant uncertainty in objectively ranking stressors, which may limit the regional synthesis of these data across the watershed. These inconsistencies across jurisdictional boundaries can originate from several factors, such as a focus on different designated uses, the initial state-specific stressor identification process, different descriptors, identification of suspected sources, and how data are submitted and/or updated within the ATTAINS database. Despite impairment waterbodies being 303(d) listed and being assigned a TMDL for impaired stressors, this updated information is not always updated in ATTAINS, and differences between state and federal databases prevent this information from being effectively harmonized and integrated. Finally, only one cause per designated use is reported on the 303(d) lists, whereas the studies in the literature review often reported multiple stressors as potential causes.

#### Limitations regarding this data analysis

Given the diversity of assessment methodologies used in the peer-reviewed literature, we limited the meta-analysis to those studies with adequate statistical analyses to describe the relative relationships between different stressors and a biological response metric. This constraint excluded some studies with study designs that did not support such analyses (e.g., biological response metrics and stressors described as a function of distance from a point source). As a result, the meta-analysis largely reflects studies with a focus on major land use categories as sources (urban, agricultural, or mining activities; Fig. 3). We also limited the stressors considered in the analysis to those that are proximal to the stream and those reported in the jurisdictional impairment analysis. As a result, several factors that could impact benthic macroinvertebrates were not included in the analysis, such as habitat connectivity and biotic factors (i.e., invasive species). The meta-analysis did not incorporate relative rank of stressors. Each study was also given equal weight regardless of study design or number of OUs. There is greater uncertainty in the meta-analysis results for stressors that were rarely measured (i.e., toxic contaminants), given there were fewer studies available from which we could draw conclusions. The jurisdictional impairment analysis was limited to those impairments designed for ecological life uses to align with the outcomes presented in the literature. Additional information on stressors is reported for other designated life uses, such as drinking water and fish and shellfish consumption, which may be relevant to ecological outcomes, yet often have different thresholds for listing (e.g., contaminant levels in fish relevant to human consumption; Fig. SI-5). Leveraging these additional datasets could provide additional information on other contaminants that could be impacting stream biological communities.

### Future Research Needs

Our analysis revealed that the effects of toxic contaminants (e.g., pesticides) and flow alteration are likely under-represented in stressor identification assessments in the region, so more work is needed to fully understand their contributions to biological impairment. Ideally, direct sampling of pesticides in stream reaches is most helpful since many factors influence the spatial distribution of pesticides in streams (Smalling et al. 2021). Pesticide monitoring can be focused on the likely types found in certain settings (Nowell et al. 2021). There have also been efforts to predict pesticide loading in streams nationally using a watershed modeling framework (Stone et al. 2013), which can be used to determine whether certain reaches might be at higher risk of impacts from pesticides. Flow alteration in urban settings can be driven by a myriad of factors, including the spatial arrangement of impervious areas in a watershed (Debbage and Shepherd 2018). As such, direct monitoring of stage or discharge, especially if coupled with rainfall monitoring (Fanelli et al. 2017), could help better characterize flow regime alteration. Recent work to predict flow alteration across stream reaches in the CB watershed can also be used to reflect the relative risk that a stream reach is experiencing flow alteration (Maloney et al. 2021). Given that flow alteration may impact other stressor categories (Fig. 1b; Table 3), better characterization of this stressor category could be helpful in prioritizing management actions. For example, flow alteration itself may facilitate transport of pesticides in urban areas (Carpenter et al. 2016) and fundamentally underpins geomorphic and bed habitat degradation.

More work is also needed to better understand the role of stressor interactions in biological impairment. The effect of multiple stressors on biological responses can be additive, which occurs when the overall effect of multiple stressors is the sum of the individual effects of each stressor; synergistic, or when the overall response is greater than the additive effects of the individual stressors; or antagonistic, when the combined effect of the stressors is less than additive (Crain et al. 2008). A recent U.S. national synthesis revealed fish, benthic macroinvertebrate, and diatom community metrics were all negatively associated with the number of stressors present in the stream, indicating at least additive or synergistic effects of multiple stressors (Waite et al. 2021). In a similar synthesis in Europe, 30% of stressor interactive effects on fish community metrics were found to be synergistic (Schinegger et al. 2016), highlighting the need to incorporate these effects in stressor identification assessments. Testing for and reporting on the effects of stressor interactions is typically done only in flume or mesocosm studies; examples included the interactive effects of pesticides, coarse sediment, and nutrients (Brooks et al. 2021), fine sediment, nutrients, and temperature (Piggott et al. 2012), and salinity, fine sediment, and flow (Beermann et al. 2018). Although these studies are quite useful to prioritize stressors, care should be taken to extrapolate these findings as these experiments are done under controlled settings and may not reflect stressor-response relationships observed in the field (Kreyling et al. 2018). Advancing our understanding of the effects of chemical mixtures will also help quantify the effects of multi-contaminant settings (see Covert et al. 2020 as an example).

Finally, adopting common terminology, conceptual understandings of source-stressor-response relationships, and study designs may also help to generalize the findings from jurisdictional stressor identification assessments and research studies across the region. Stressor identification assessments use a variety of terms to describe what are defined as sources, stressors, and their relationships (Orr et al. 2020), making comparisons across studies difficult. Some jurisdictions already leverage concepts from EPA’s Causal Analysis/Diagnosis Decision Information System (CADDIS; US EPA 2017), which is a weight-of-evidence approach to stressor identification. This resource provides standard “source-to-impairment” conceptual diagrams outlining likely pathways between sources (urbanization, energy extraction), proximate stressors, modes of action, and ecosystem responses. EPA’s CADDIS could be a good starting point for unifying key terms and conceptual frameworks for stressor identification and could reduce the likelihood of attributing biological impairment to a proxy or co-occurring stressor. Leveraging common study designs that are more likely to provide evidence needed to support stressor identification is also necessary (Orr et al. 2020). For example, studies following stressor gradients facilitate the identification of ecological thresholds for individual stressors (Richards et al. 2018). Complicating these studies, however, is often the presence of multiple stressors along the same gradient (for example, Yuan et al. 2003) and more work is needed to explicitly test for stressor interactions in gradient-based studies (Kreyling et al. 2018).

## Conclusions

There is great interest by natural resource managers to have a better understanding of the primary stressors impacting freshwater ecosystems to prioritize monitoring and management activities. This is especially true in the Chesapeake Bay watershed, USA, where efforts are underway to maintain or improve the biological integrity of freshwater streams and rivers. Our analysis revealed the suite of stressors reported to be acting on benthic macroinvertebrate community metrics varied across different settings and among jurisdictions. Overall, geomorphology is likely a regional primary stressor, especially in agricultural settings, whereas salinity and major ions is likely a regional primary stressor in urban and mining settings. Toxic contaminants are likely a primary stressor where sources exist. Most impairment listings for toxic contaminants in the region are from legacy sources (e.g., acid drainage from mining), and more information is needed about contemporary sources of contaminants, such as residential and agricultural pesticide use. Flow alteration is also under-studied and rarely characterized despite its importance for influencing other prominent stressors, such as geomorphology and sediment dynamics. There were some limitations in our analysis — for example, the diversity of study design and analysis approaches employed by the multi-stressor studies limited their inclusion in the meta-analysis. The analysis also revealed clear distinctions between jurisdictional approaches to stressor identification. Despite these limitations, this study improved our understanding of the major stressors likely contributing to stream ecosystem biological impairment in the CB watershed.

## Supplementary Information

Supplemental Information

## Data Availability

All data used in the analysis is available in a USGS data release (Fanelli and Cashman 2022).
