# Correction to: Identifying Key Stressors Driving Biological Impairment in Freshwater Streams in the Chesapeake Bay Watershed, USA

**DOI:** 10.1007/s00267-022-01740-6

**Published:** 2022-10-31

**Authors:** Rosemary M. Fanelli, Matthew J. Cashman, Aaron J. Porter

**Affiliations:** 1 U.S. Geological Survey, South Atlantic Water Science Center, Raleigh, NC USA; 2 U.S. Geological Survey, Maryland-D.C.-Delaware Water Science Center, Baltimore, MD USA; 3 U.S. Geological Survey, Virginia-West Virginia Water Science Center, Richmond, VA USA

Correction to: *Environmental Management* 10.1007/s00267-022-01723-7

In this article, the designations a, b and c were missing on the individual panels in Figure 4. The Figure 4 has now been corrected.

The original article has been corrected.

