# Supplementary material for: Identifying Key Stressors Driving Biological Impairment in Freshwater Streams in the Chesapeake Bay Watershed, USA
Source: Environ Manage. 2022 Oct 7;70(6):926–49. doi: 10.1007/s00267-022-01723-7 (PMC9622507; doi:10.1007/s00267-022-01723-7)
Supplement: Supplementary file 1 — Supplemental Information [file 267_2022_1723_MOESM1_ESM.docx]

**Supplemental Information** for “Identifying key stressors driving biological impairment in freshwater streams in the Chesapeake Bay watershed”

Authors: Rosemary Fanelli^1*^, Matthew Cashman^2^, and Aaron Porter^3^

^1^U.S. Geological Survey, South Atlantic Water Science Center, Raleigh, NC

^2^U.S. Geological Survey, Maryland-D.C.-Delaware Water Science Center, Baltimore, MD

^3^U.S. Geological Survey, Virginia-West Virginia Water Science Center, Richmond, VA

*Corresponding author, email: [rfanelli@usgs.gov](mailto:rfanelli@usgs.gov)

Additional details on methodology

**Study eligibility for literature review meta-analysis:**  The data release associated with this publication (Fanelli and Cashman 2022) contains a literature review summary table, which includes general study information (first author, title, year published, journal); study design information (geographic location, sources studied, stressors measured) and whether it was included in the meta-analysis. The column, “Paper_decision_code”, describes eligibility for inclusion in the meta-analysis. Assigned codes include: 1 = eligible and included; 2 = not eligible, study only assessed one stressor; 3 = not eligible, benthic macroinvertebrates were not included as a response variable; 4 = not eligible, no in-stream stressors measured in study; 5 = not eligible, no direct analysis of stressor and biological response datasets; 6 = not eligible due to insufficient statistical analysis and/or reporting; and 7 = not eligible, out of scope of the study. In general, studies were eligible if they employed some type of variable selection analysis, such as multiple linear regression or random forests, to determine the relative strength of a stressor to explain variability in the response variable. We did include studies that reported results from simple correlation analyses if all stressors were included in the analysis. Studies with no direct analysis of stressor biological datasets (category 5 for exclusion) were often point source studies for which changes in stressors and biological responses were described and/or quantified with respect to distance from a point source (see Echols and others 2009 as an example). Studies with insufficient statistical analysis and/or reporting (category 6 for exclusion) were often small studies that were more descriptive, and whose goals were to describe overall spatial differences among sites (see Roy and others 2018 as an example).

Citation and link for the data release can be found here:

Fanelli R.M. and M. J. Cashman. 2022. Literature review results and regulatory summaries of freshwater stressors influencing biological impairment in the Chesapeake Bay watershed, USA. U.S. Geological Survey Data Release. <https://doi.org/10.5066/P9DHJSLF>.


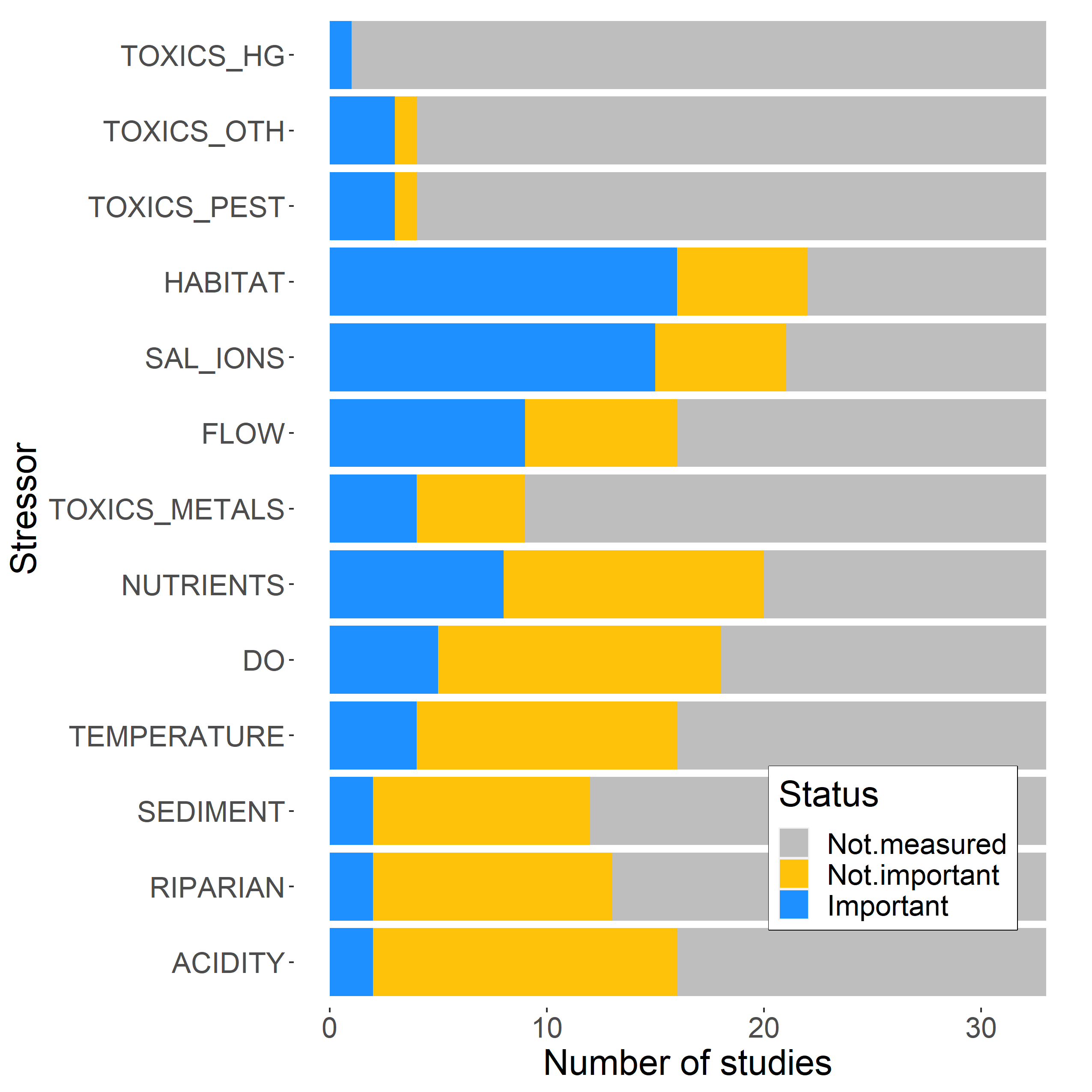

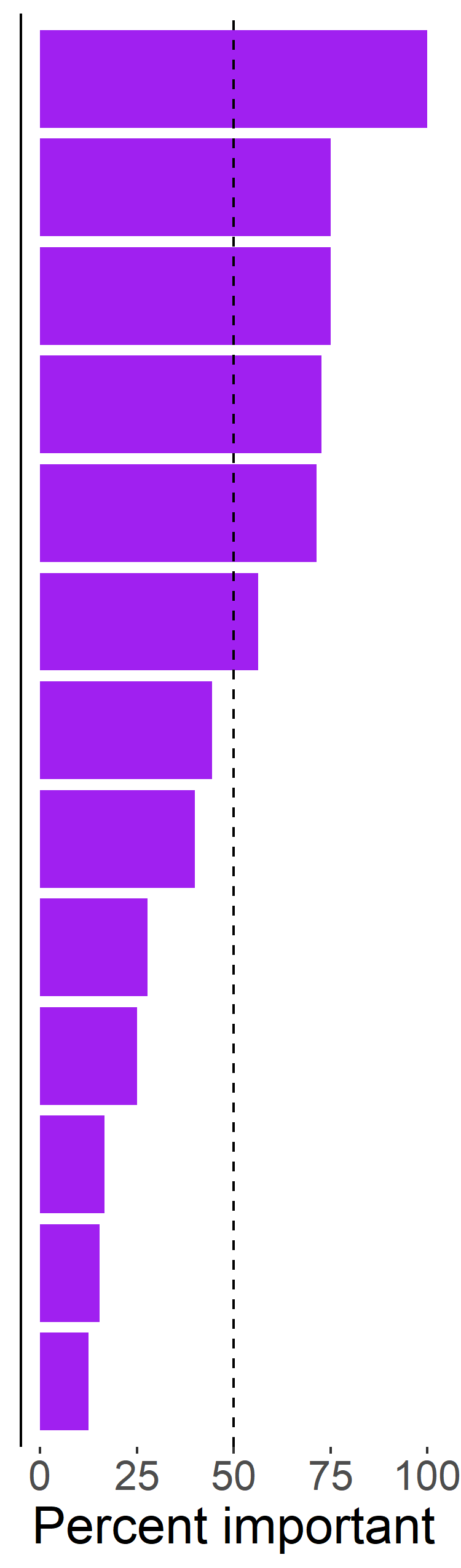


**Fig. SI-1** Barplot showing number of studies (x-axis) that measured a fine-scale stressor category and reported it as important for explaining biological responses (left), as well as the percent of studies which measured the stressor also reporting it as important is shown on the (right) for all studies in the meta-analysis (all studies, n=33). The geomorphology major stressor category is subdivided into two finer categories (“SEDIMENT” and “HABITAT”, see methods section for more details). The toxic contaminants major stressor category is subdivided into four finer categories: mercury, metals, pesticides, and other organic contaminants (“TOXICS_HG”, “TOXICS_METALS”, “TOXICS_PEST”, and “TOXICS_OTH”, respectively). Vertical dashed line on right denotes 50%.  SAL_IONS = Salinity and other ions. DO = dissolved oxygen.


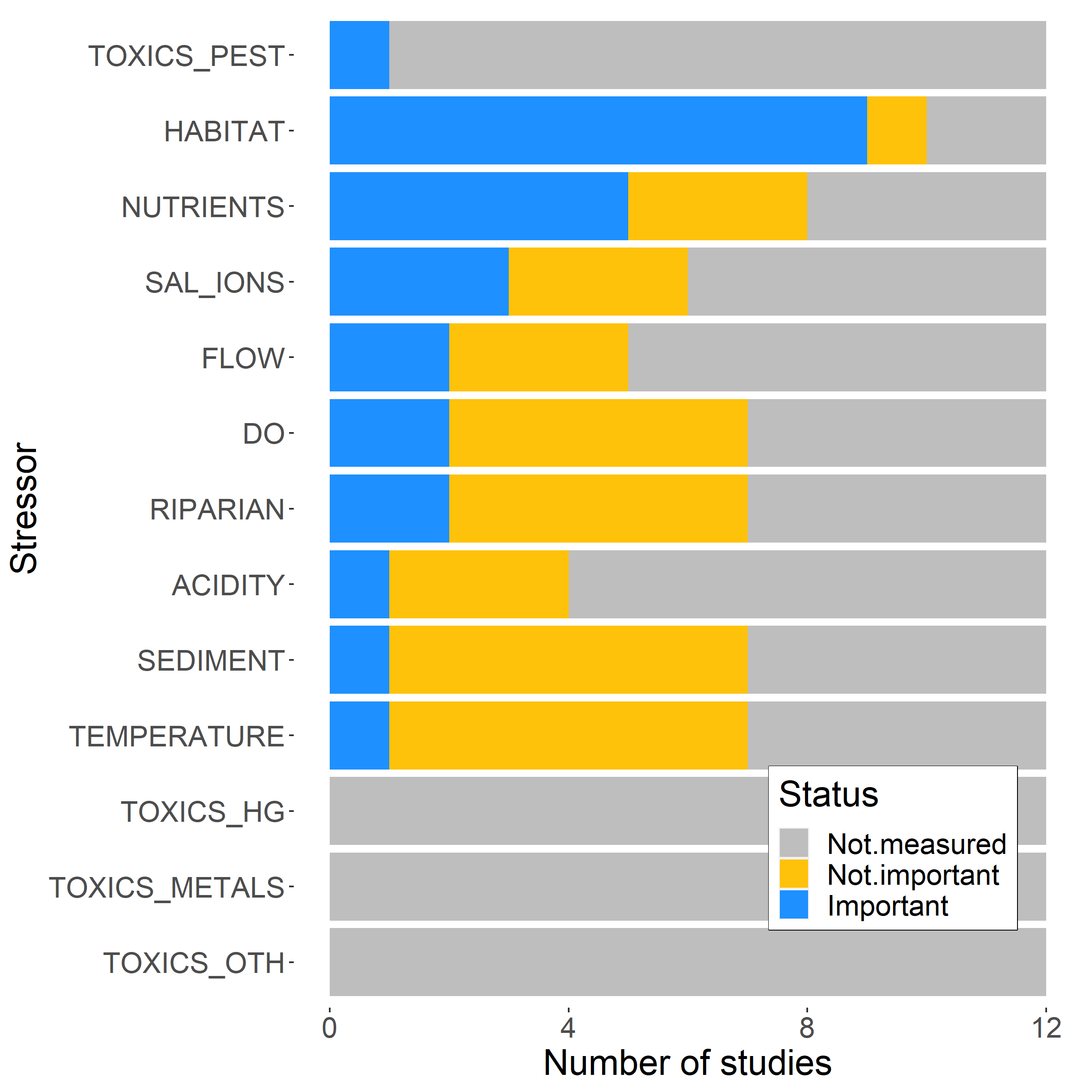

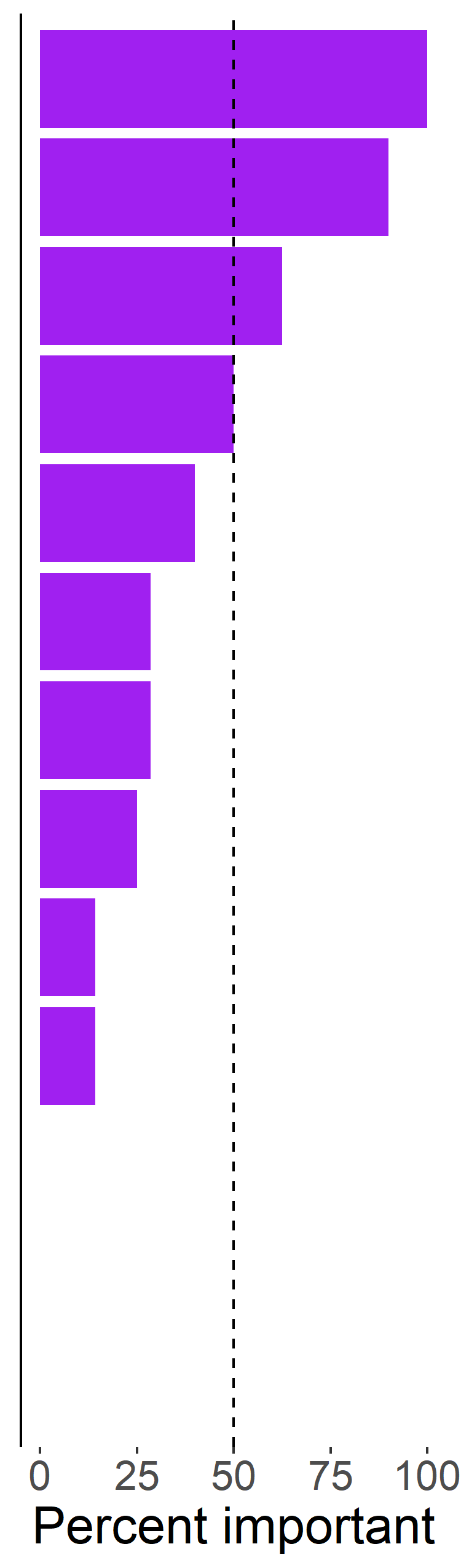


**Fig. SI-2** Barplot showing number of studies (x-axis) that measured a fine-scale stressor category and reported it as important for explaining biological responses (left), as well as the percent of studies which measured the stressor also reporting it as important is shown on the (right) for agricultural studies in the meta-analysis (n=12). The geomorphology major stressor category is subdivided into two finer categories (“SEDIMENT” and “HABITAT”, see methods section for more details). The toxic contaminants major stressor category is subdivided into four finer categories: mercury, metals, pesticides, and other organic contaminants (“TOXICS_HG”, “TOXICS_METALS”, “TOXICS_PEST”, and “TOXICS_OTH”, respectively). Vertical dashed line on right denotes 50%. SAL_IONS = Salinity and other ions. DO = dissolved oxygen.


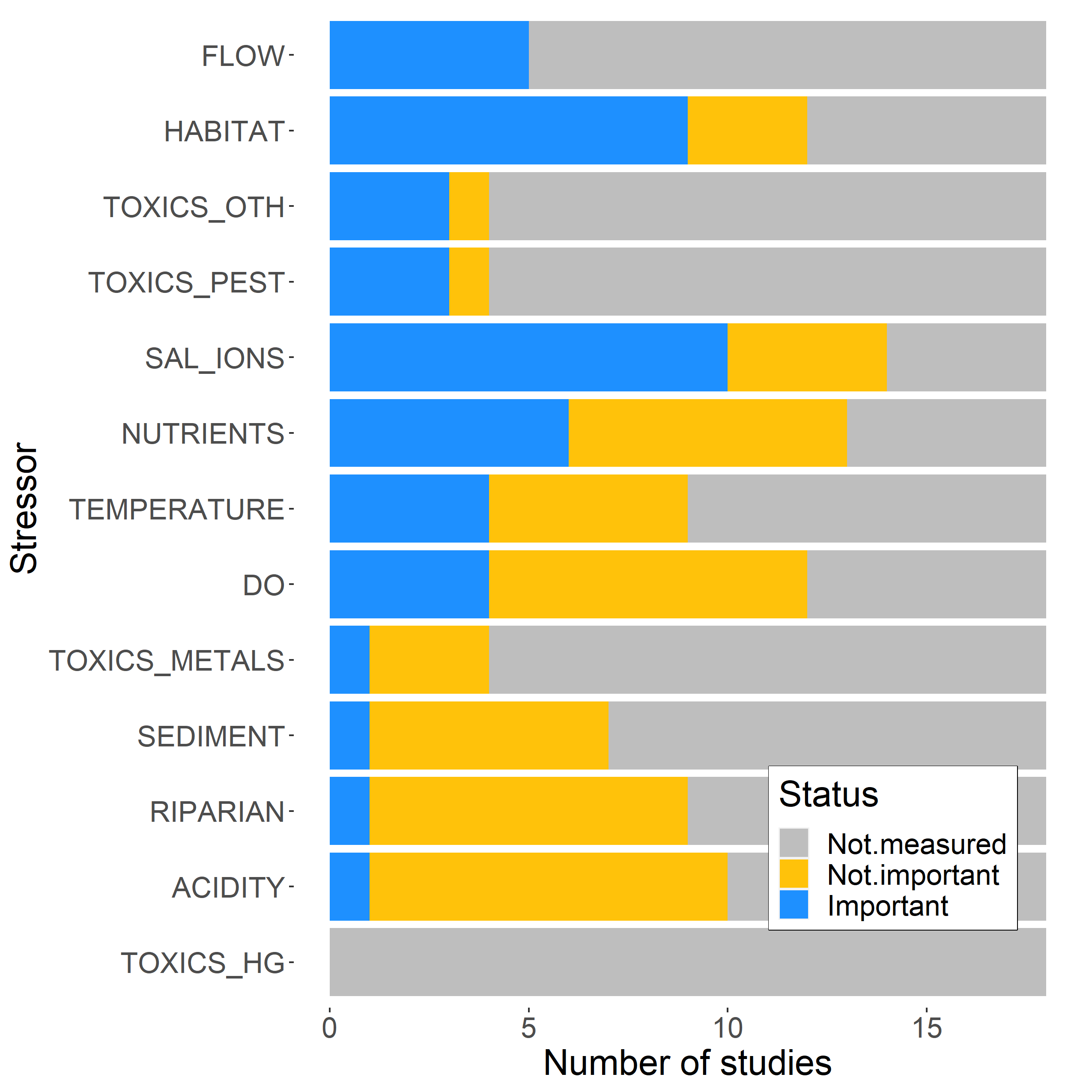

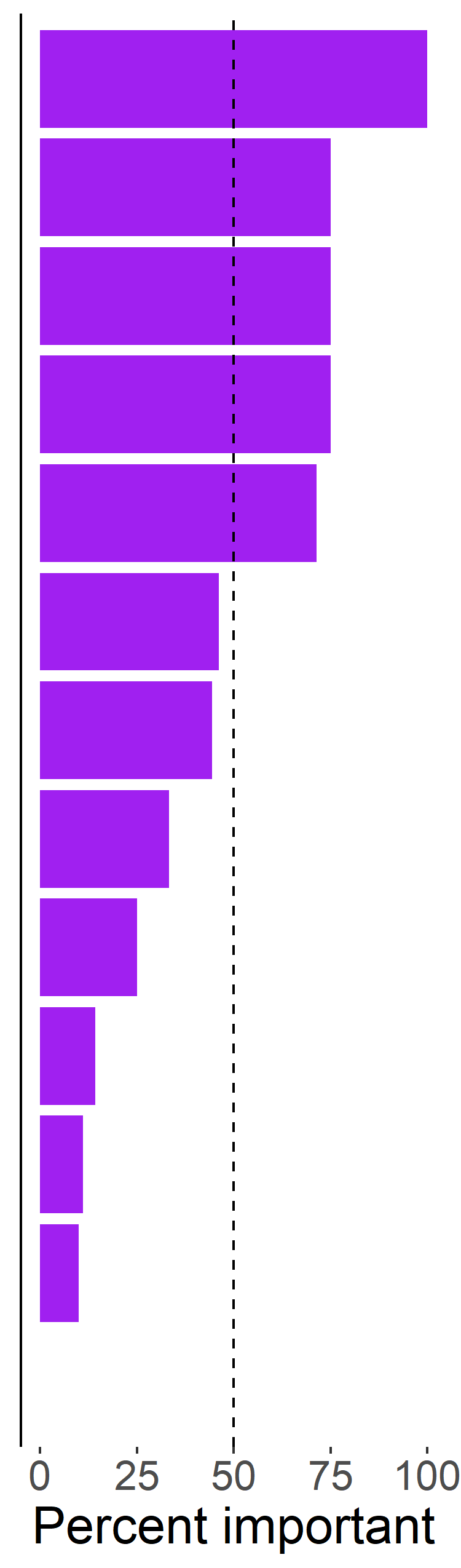


**Fig. SI-3** Barplot showing number of studies (x-axis) that measured a fine-scale stressor category and reported it as important for explaining biological responses (left), as well as the percent of studies which measured the stressor also reporting it as important is shown on the (right) for urban studies in the meta-analysis (n=18). The geomorphology major stressor category is subdivided into two finer categories (“SEDIMENT” and “HABITAT”, see methods section for more details). The toxic contaminants major stressor category is subdivided into four finer categories: mercury, metals, pesticides, and other organic contaminants (“TOXICS_HG”, “TOXICS_METALS”, “TOXICS_PEST”, and “TOXICS_OTH”, respectively). Vertical dashed line on right denotes 50%. SAL_IONS = Salinity and other ions. DO = dissolved oxygen.


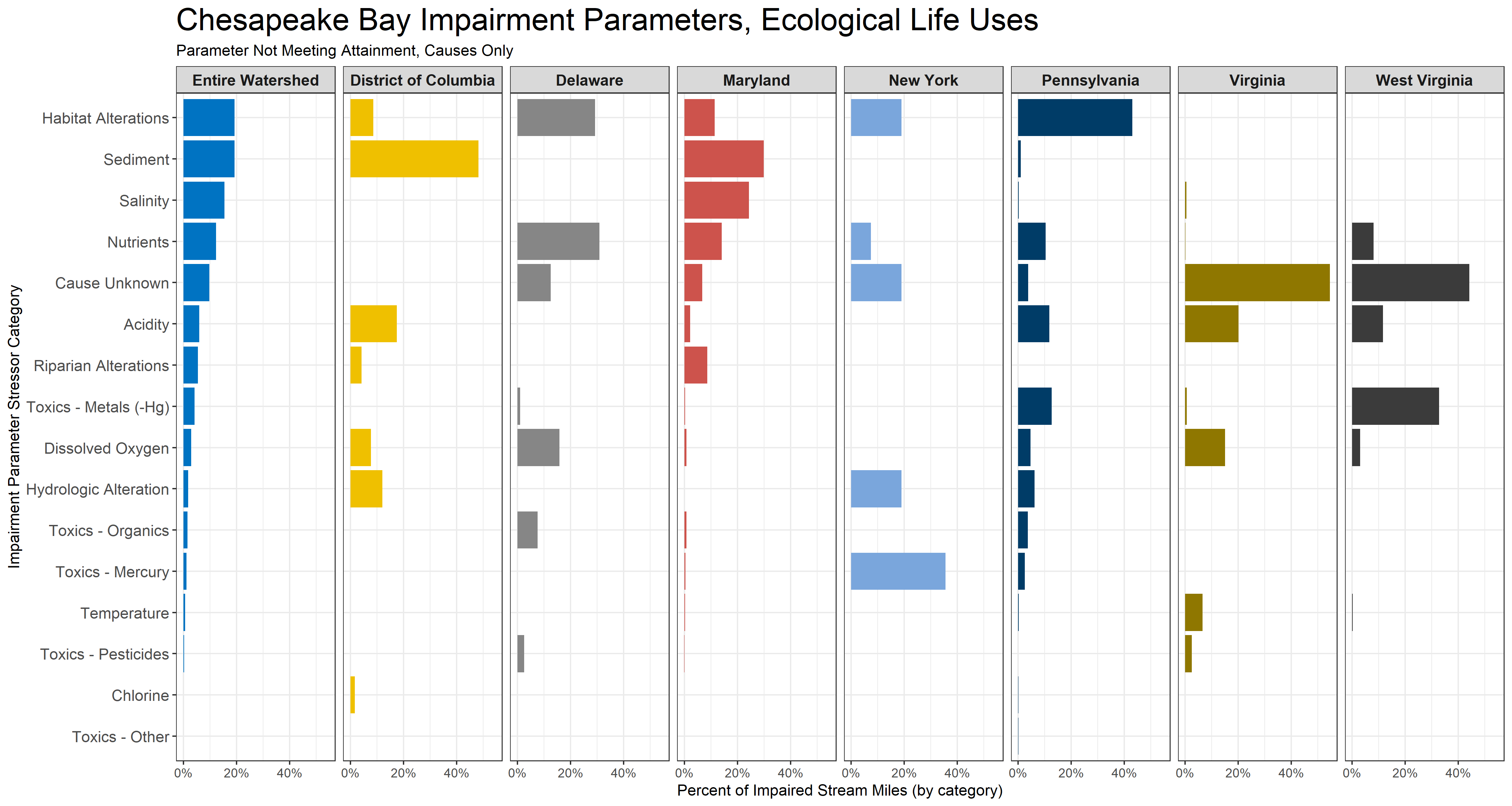


**Fig. SI-4** Percent of all impaired stream miles causally attributed to a stressor category within each jurisdiction, using fine stressor categories from the jurisdictional impairment analysis (Ecological Life designated uses only). The geomorphology major stressor category is subdivided into two finer categories (“Sediment” and “Habitat alterations”). The toxic contaminants major stressor category is subdivided into finer categories: mercury, metals, pesticides, other organic contaminants, other non-organic contaminants, and chlorine (“Toxics – Mercury”, “Toxics – Metals (-Hg)”, “Toxics - Pesticides”, “Toxics - Organics”, “Toxics – Other”, and “Chlorine”, respectively).


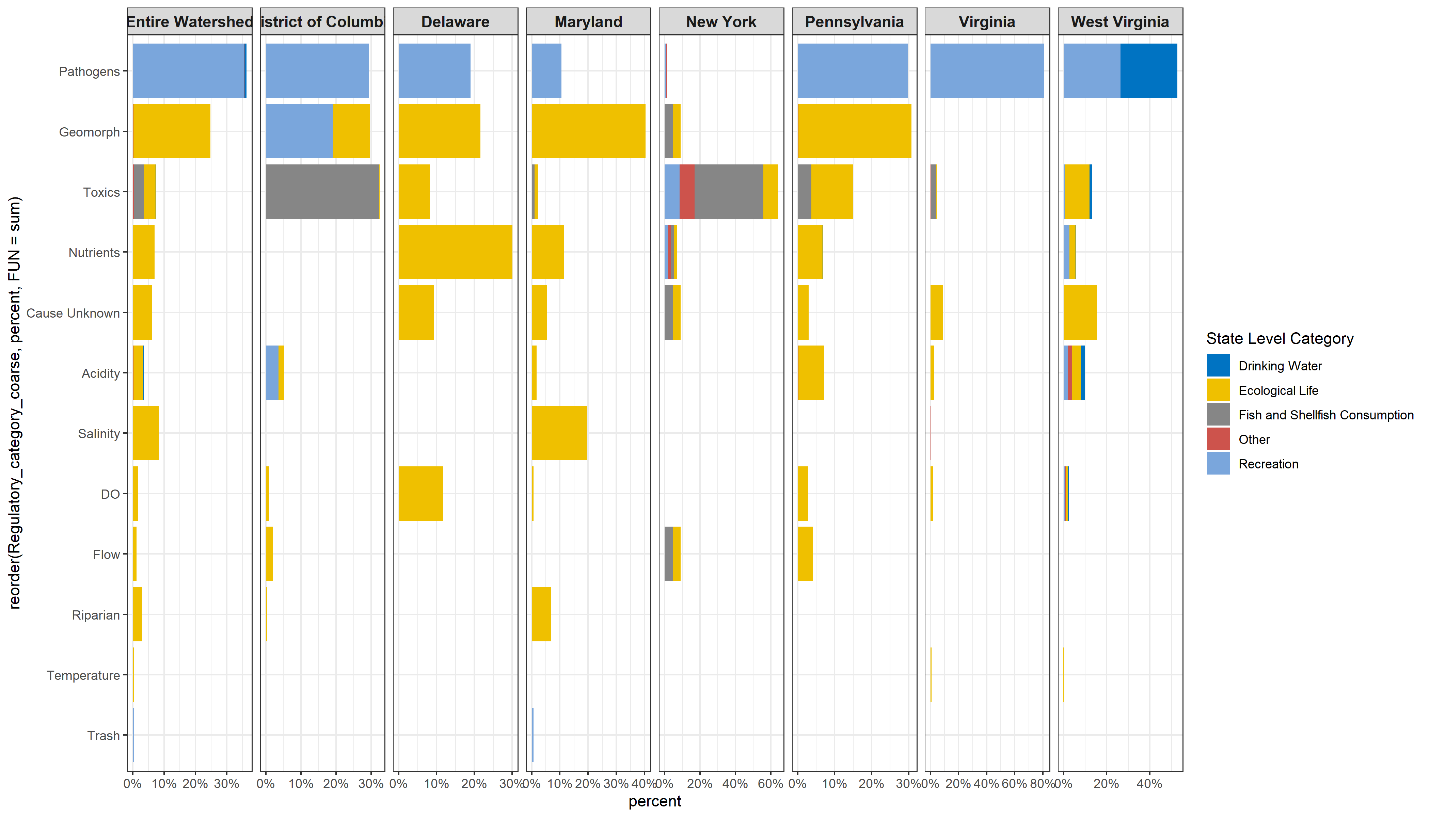


**Fig. SI-5** Percent of all impaired stream miles causally attributed to a stressor category within each jurisdiction, using coarse stressor categories from the jurisdictional impairment analysis for all designated life uses reported in the U.S. EPA’s Assessment, Total Maximum Daily Load Tracking and Implementation System (ATTAINS) database. State level category = designated life use for the water body. Two additional coarse stressor groups are included in this figure- pathogens and trash - as they are reported as causes for impairment for life uses other than ecological life uses (i.e., recreation or drinking water).
